# Hidden state inference requires abstract contextual representations in ventral hippocampus

**DOI:** 10.1126/science.adq5874

**Published:** 2024-11-21

**Authors:** Karyna Mishchanchuk, Gabrielle Gregoriou, Albert Qü, Alizée Kastler, Quentin J. M. Huys, Linda Wilbrecht, Andrew F. MacAskill

**Affiliations:** 1 Department of Neuroscience, Physiology and Pharmacology, University College London; Gower St, London, WC1E 6BT; 2 Helen Wills Institute of Neuroscience, Department of Psychology, University of California; Berkeley, CA, 94720; 3 Applied Computational Psychiatry Lab, Mental Health Neuroscience Department, Division of Psychiatry and Max Planck UCL Centre for Computational Psychiatry and Ageing Research, Queen Square Institute of Neurology, University College London; 10-12 Russell Square, London, WC1B 5EH

## Abstract

The ability to utilize subjective, latent contextual representations to influence decision making is crucial for everyday life. The hippocampus is hypothesized to bind together otherwise abstract combinations of stimuli to represent such latent contexts, to support the process of hidden state inference. Yet, evidence for this remains limited. We found that the ventral hippocampus is required for mice to perform hidden state inference during a 2-armed bandit task. Hippocampal neurons differentiate the two abstract contexts required for this strategy in a manner similar to the differentiation of spatial locations, and their activity is essential for appropriate dopamine dynamics. These findings offer insight into how latent contextual information is used to optimize decisions and emphasize a key role for the hippocampus in hidden state inference.

Animals (and humans) have a remarkable capacity for maintaining optimal behavior when faced with a constantly changing environment. This can be done in two main ways: 1) a subject can presume varying outcomes occur in a single context: if an action is rewarded, the subject can increase the likelihood of repeating it, and similarly decrease the likelihood if unrewarded – a common implementation of this is called ‘Q-learning’. 2) a subject can instead learn that the outcome associated with particular action can differ dependent on the context it takes place in. While Q-learning can perform well when such contexts are unambiguous and fully signaled, context is often hidden and must be inferred - requiring the use of what is often called ‘hidden state inference’. While there is much understanding of how outcome-focused information can be used to guide behavior when the context is unambiguous, the neural basis of ‘hidden state inference’ over multiple contexts remains unclear.

The process of hidden state inference is thought to be achieved by processing otherwise stochastic and volatile information into stable latent representations (1–3). Once learnt, these subjective representations can be used to infer the most likely context in an otherwise ambiguous situation, and thus guide optimal behavior (4–10). Deficits in hidden state inference are a feature of many of the most debilitating neural disorders (11–15). However, the means by which such subjective, latent contexts are represented and utilized in the brain to support hidden state inference remains unclear.

The hippocampus organizes sensory stimuli into representations of discrete spatial locations and contexts to guide flexible behavior (16–18). This has led to the hypothesis that hippocampal circuitry, and in particular more ventral parts of hippocampus commonly associated with affective processing, may play a crucial role in hidden state inference more generally – through the creation and utilization of subjective, often non-spatial latent contexts (1, 3, 19–26). However, there is little direct investigation of how the hippocampus represents such information at the neural level, and how this might support behavior.

We used a ‘2-armed bandit’ task in mice - a commonly used probabilistic serial reversal learning task (9, 12, 27–30). Subjects are presented with a choice of two levers, each with different probabilities of reward, and must use trial and error to identify the high probability choice. After a variable number of correct choices – unsignaled to the subject – the contingencies of the levers switch. Therefore, subjects must both identify and exploit the current high probability lever, but also be able to rapidly switch their choice after a switch in contingencies.

To solve this task mice can either use Q-learning – where they incrementally update the value associated with each of the two actions, based on the fluctuating outcomes; or alternatively, mice can use hidden state inference – where they form a representation of the two latent contexts that make up the task, and use this to guide behavior (4, 9, 12). In such reversal learning tasks, a state inference strategy will often result in a higher reward rate (4, 8, 9).

## An operant 2-armed bandit task for mice

**Fig. 1:**
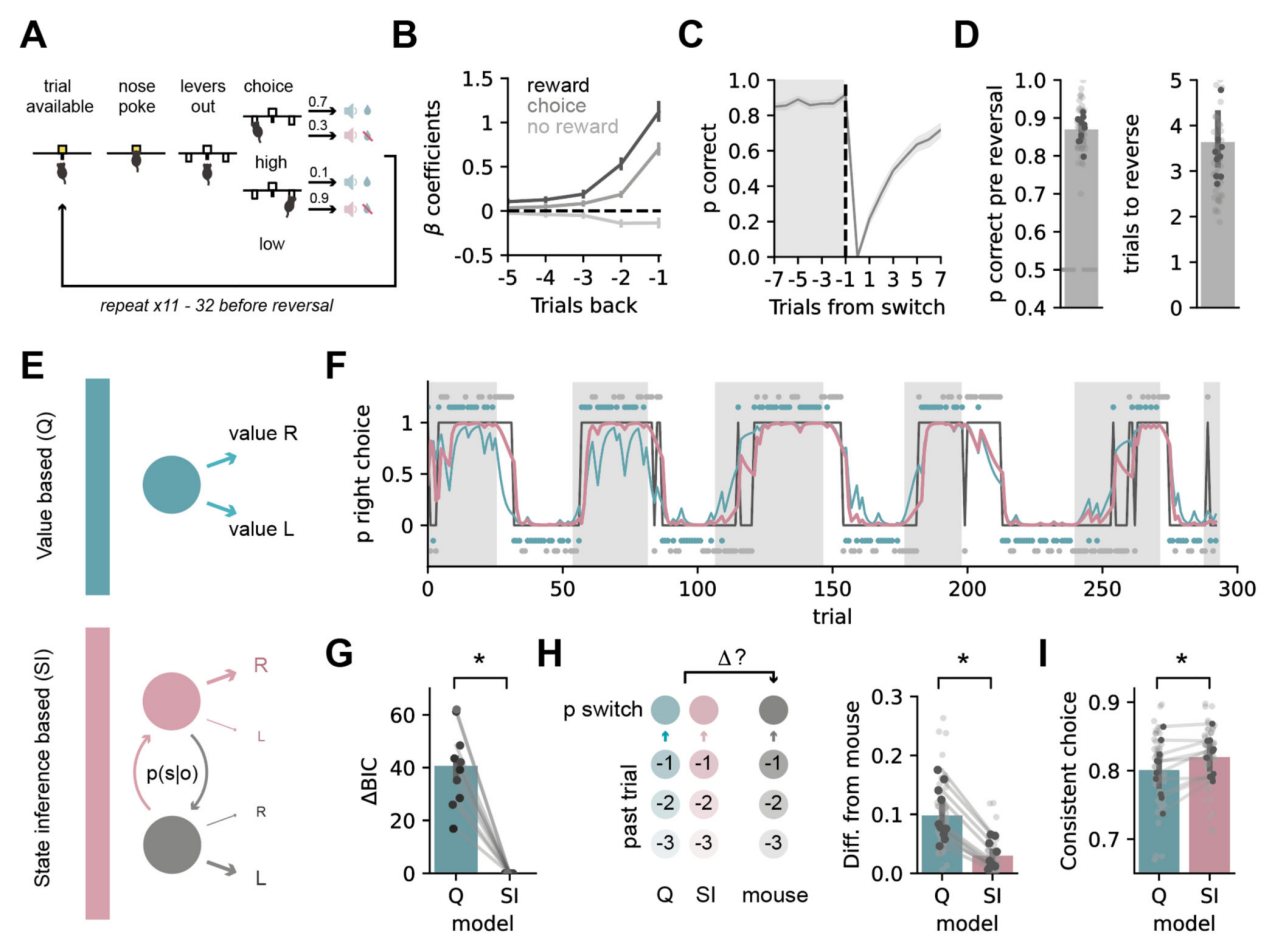
Mice utilize a contextual, state inference strategy to solve a 2-armed bandit task. Schematic of the task. (**B)** Regression coefficients from a logistic regression model predicting animal’s choice on a given trial based on rewarded choice (black), unrewarded choice (light grey) or outcome-independent choice (grey) history. (**C)** Average proportion of high probability choices around a switch in contingency. (**D)** Left, proportion of high probability choices over the 8 trials before the block reversal. Right, number of trials to switch to the new high probability choice following block reversal. **(E)** Schematics of Q and SI model strategies. (**F)** Example behavioral session. Grey boxes show blocks of the right lever being the high probability choice; black trace shows animals’ choices (top = right lever press, bottom = left lever press); probability of right choices predicted by the Q (blue) or SI (pink) models are superimposed; blue and grey dots at top and bottom show rewarded and non-rewarded trials. (**G)** Summary of model fits to Q and SI models using Bayesian Information Criterion. Difference in BIC score from most parsimonious model was calculated for each session and averaged across mice. (**H)** Summary of difference between mouse switching behavior at different trial histories, and switch probability estimates for the same choice and outcome histories from either Q or SI strategies. See full trial history analysis in fig. S1 (**I)** Proportion of choices consistent with a Q or SI strategy. Dark points show individual mice, light points show individual sessions. Error bars represent s.e.m. across animals (n = 10). *P < 0.05.

To examine the representation of subjective, latent contexts, we implemented an operant version of a 2-armed bandit task (Fig. 1A) (9, 27, 28). Mice initiated a trial with a nose poke, and were then presented with two retractable levers either side of the nose port. Pressing one of these levers resulted in reward (6 µl of 10 % sucrose solution) accompanied by an auditory cue with 70 % probability (high-probability lever), while pressing the opposite lever was rewarded with 10 % probability (low-probability lever). The reward contingencies reversed after 10 to 32 high-probability lever choices.

Following several stages of training (see Methods), mice reached high performance, and used a combination of past rewards and choices to guide their behavior (Fig. 1B, C). Mice could closely track the identity of the high-probability lever (~90% correct at the end of each contingency block) and quickly captured the changes in the reward contingencies (choosing the new lever above chance ~4 trials after a switch in contingency, Fig. 1D).

## Mice utilize a state inference strategy to perform a 2-armed bandit task

We asked what strategy best described mouse behavior during the task. We focused on a comparison between Q-learning agents (Q) that presume only one context; and state inference (SI) agents that infer the probability of being in one of two latent contexts, and use this to estimate the optimal choice (Figure 1E) (8, 9, 29, 31). We also performed a comprehensive comparison of the most commonly utilized models (fig. S1-3), which allowed us to identify the most appropriate Q and SI agents for further analysis (see Methods).

Across all sessions and all mice models utilizing SI consistently best described mouse behavior. We found equivalent results using several complementary metrics. First, we used Bayesian Information criterion (BIC) to identify the most parsimonious model (Fig. 1G). Second, the probability of a mouse switching its choice was strongly shaped by different choices and their outcomes on the past three trials (9,30) (Fig. 1B, fig. S1). This pattern of switching behavior was well recapitulated by switch probability estimates from SI, but not Q models (Fig. 1H). Finally, we looked at trial-to-trial model predictions and found that mice were more likely to choose the option predicted by an SI rather than a Q strategy (Fig. 1I).

## Dopamine dynamics incorporate state inference during task performance

Our results so far suggest that mice utilize state inference to guide behavior in the 2-armed bandit task. Reward prediction errors (RPE) signaled by dopamine release in the nucleus accumbens core (NAc) strongly incorporate predictions from SI, and such signals are increasingly used as a functional readout of the use of SI strategies (6, 7, 9, 32).

**Fig. 2:**
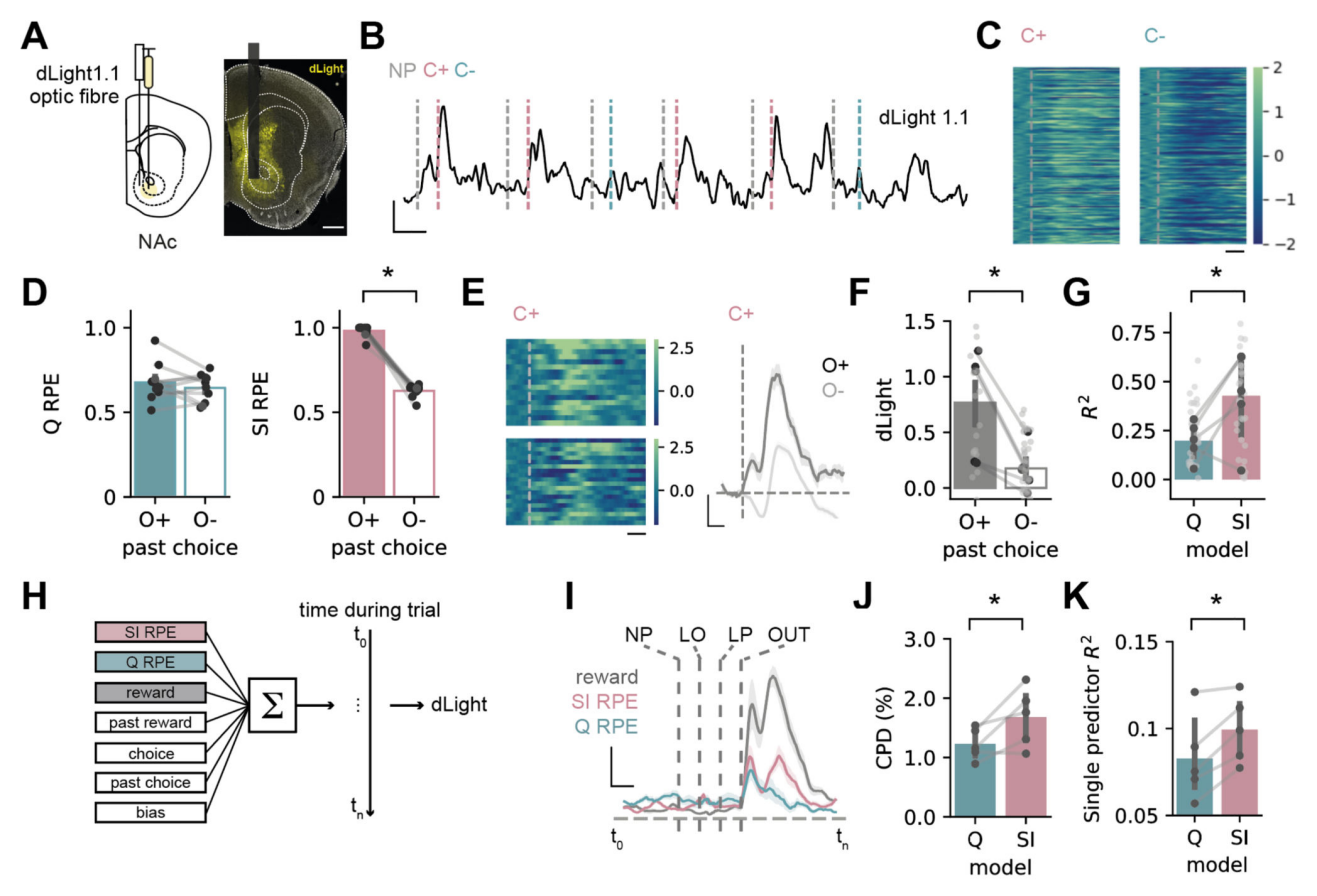
Dopamine in NAc core reflects use of a state inference strategy. Schematic of dLight1.1 injection and fiber placement in nucleus accumbens core (NAc). Scale bar = 400 μm. (**B**) Example recording during task. Grey lines indicate trial initiation, pink lines indicate reward delivery with auditory cue (C+), and blue lines indicate unrewarded cue (C-). Scale bar = 2 Z, 5 seconds. (**C**) Z-scored dLight signal time-locked to C+ or C-. Scale bar = 1 s. (**D**) Prediction error estimates from Q (left) or SI (right) model simulations for trials split according to outcome of past opposite lever press (see text): O+ (reward) or O- (reward omission). (**E)** Left, z-scored dLight signal aligned to C+ for O+ (top) or O- (bottom) trials. Right, example z-scored dLight signal around C+ for O+ and O- trials for one session. Scale bar = 1 s (left); 1s, 0.5 zF (right). (**F**) Summary of C+ dLight signal on O+ and O- trials. (**G**) Variance across different past choice and outcome combinations explained by prediction error estimates from either Q or SI models. (**H**) Schematic outlining time-lagged ridge regression approach. (**I**) Coefficients of partial determination for outcome (grey), Q-RPE (blue) and SI-RPE (pink) at each timepoint. Scale bar = 1 s, 2.5 % (**J**) Mean coefficient of partial determination around outcome for Q-RPE and SI-RPE. (**K**) Variance explained by Q-RPE or SI-RPE predictors alone. Dark points show individual mice, light points show individual sessions. Error bars represent s.e.m. across animals (model simulations n = 10; dopamine recordings n = 5). *P < 0.05.

We hypothesized that NAc dopamine would contain equivalent signatures of SI during performance of the 2-armed bandit. We expressed the extracellular dopamine sensor dLight1.1 (33) in NAc core (Fig. 2B, C). We then used two complementary strategies to show that there was strong and consistent influence of SI on NAc dopamine during task performance.

First, we took advantage of the fact that Q and SI-based strategies have different mechanics. The Q based agent updates the value of an individual choice only when it is chosen. Therefore, for a given choice, Q-RPE is influenced by a past outcome only if that outcome was associated with the same choice. In contrast, in the SI-based agent the probability of being in a particular latent context is estimated from the past outcome of both choices. Therefore SI-RPE will be influenced by past outcomes from both the same and the opposite choice (see methods and fig. S2, 3 and 5 for further analysis and discussion of how alternate models including Q-learning agents supplemented with counterfactual updating, forgetting and dynamic learning rates behave in such situations).

We isolated rewarded trials where mice chose a different lever than in the previous trial. We then separated these trials dependent on whether the past choice on the opposite lever was rewarded (O+) or not rewarded (O-). Consistent with the use of an SI model, dopamine was very different across these two trial histories, and was consistent with estimated RPE from simulations utilizing an SI strategy, but not a Q based strategy (Fig. 2D-F). We then expanded this approach, and compared all possible combinations of trial histories across the same (S) and opposite (O) levers. We found that across all trial types, dopamine was well predicted by SI RPE, and poorly predicted by Q RPE (Fig. 2G). In particular during periods of the same choices (S+ and S-) there was no large prediction error despite occasional non rewarded trials (fig. S4), consistent with the probabilistic context being known to the animal.

Second, we used regression to express dLight fluorescence as a sum of responses related to outcome, past outcome, choice and past choice, as well as trial-by-trial estimates of Q-RPE and SI-RPE (Fig. 2H). Across mice, model weights related to SI-RPE were large, especially around outcome, and consistently explained more variance in dLight signal compared to Q-RPE (Fig. 2I, J). Moreover, a greater proportion of dLight activity could be explained by SI-RPE alone, when compared to Q-RPE (Fig. 2K).

## Ventral CA1 is required for optimal performance in the 2-armed bandit task

Based on our analysis, during performance of the 2-armed bandit task, mice form latent contextual representations that are then used to optimally guide behavior and drive dopamine dynamics. We used lesions of vCA1 to test our hypothesis that the hippocampus plays a crucial role in utilizing these subjective, non-spatial latent contexts.

**Fig. 3:**
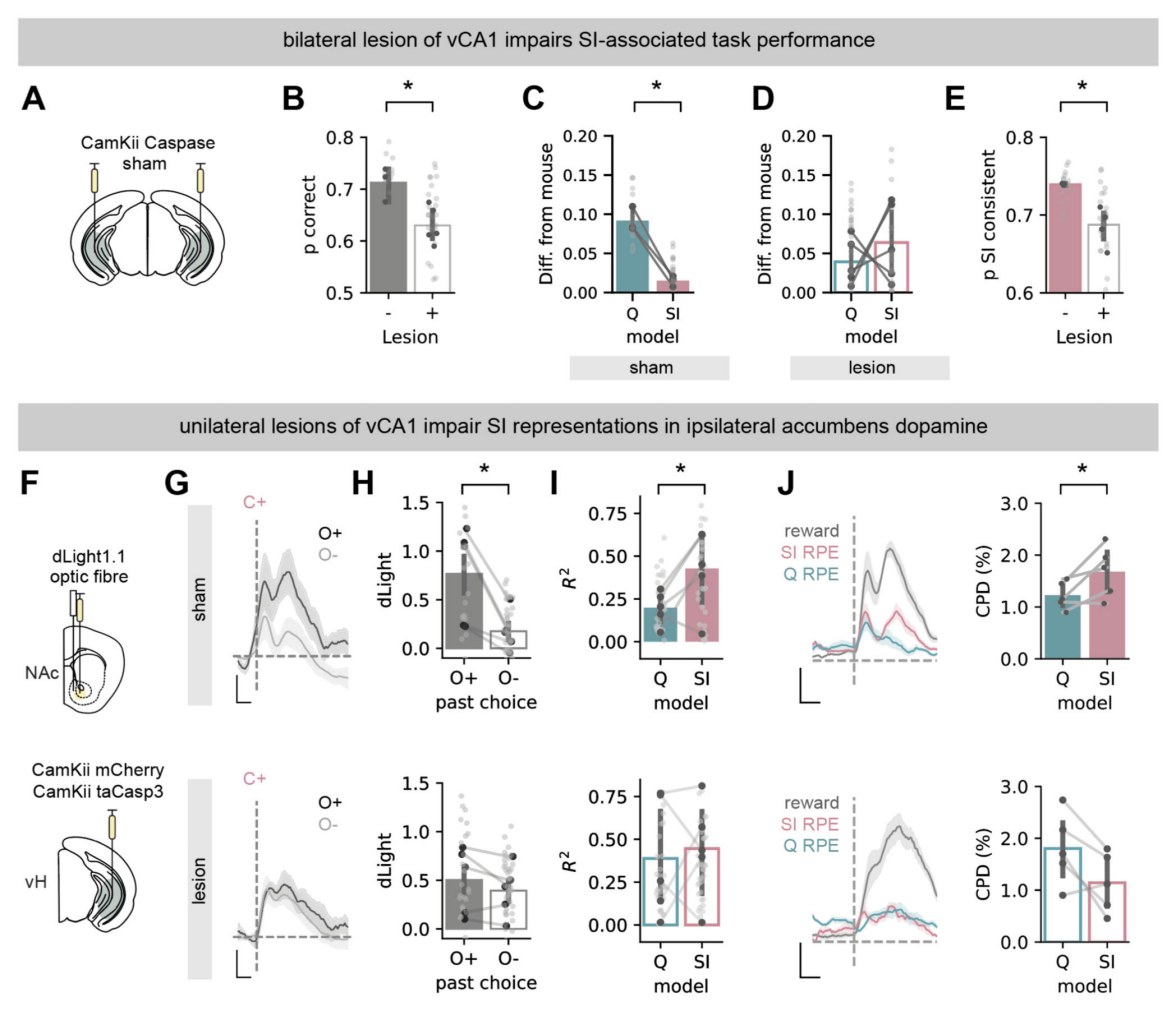
vCA1 is necessary for the neural and behavioral use of state inference Schematic of injections to express taCasp3 bilaterally in vCA1 pyramidal neurons. (**B**) Summary of high probability choices in sham and caspase lesioned mice. (**C**) Difference between mouse switching behavior at different trial histories, and that of simulations utilizing either Q or SI strategies in sham mice. (**D**) As in (**C**), but for mice with vCA1 lesions. **(E**) Proportion of choices consistent with SI strategy in sham and lesioned mice (sham n = 3, lesion n = 5). (**F**) Schematic of injections to express taCasp3 construct or control fluorophore mCherry in excitatory vCA1 neurons, and dLight1.1 and optic fiber in NAc. **G**. Mean z-scored dLight signal in sham (top) and lesion (bottom) mice, aligned to CS+, and split by different outcomes following opposite lever press in the previous trial (as in Fig. 1 K-L). Scale bar = 1s, 0.5 zF. (**H**) Summary of CS+ dLight in (**G**) in sham (top) and lesion (bottom) mice. (**I**) Variance across different past choice and outcome combinations explained by prediction error estimates from either Q or SI models in sham (top) and lesion (bottom) mice. (**J**) Left, coefficients of partial determination for outcome (grey), Q-RPE (blue) and SI-RPE (pink) at each timepoint around cs in sham (top) and lesion (bottom) mice. Right, mean coefficient of partial determination around outcome for Q-RPR and SI-RPE in sham (top) and lesion (bottom) mice. Scale bar = 1 s, 2.5 %. Dark points show individual mice, light points show individual sessions. Error bars represent s.e.m. across animals (sham n = 5, lesion n = 5). *P < 0.05.

We used bilateral expression of caspase under the control of the CaMKii promoter in fully trained mice, to ablate excitatory pyramidal neurons in vCA1 (Fig. 3A). vCA1 lesions impaired SI-associated behavior, but left behavior guided by other strategies largely intact.

When compared to sham injected controls, lesion of vCA1 impaired overall behavior (Fig. 3B). Similar to our findings in Fig. 1, the probability of a mouse switching its choice was shaped by different past choices and their outcomes in both sham and lesion groups, particularly in trials with a previous positive outcome (fig. S6). However, while the pattern of switching behavior in sham animals was well recapitulated by SI simulations, and not Q simulations (Fig. 3C), this was not apparent in lesioned animals (Fig. 3D). This was due to a decrease of the SI strategy being able to describe switching behavior in lesioned animals, accompanied by an increase in the ability of the Q strategy, with minimal changes in other strategies (fig. S6). Looking at trial-to-trial model choice probability estimates, consistent with this there was a marked decrease in the proportion of choices consistent with an SI strategy (Fig. 3E), particularly around switches in contingency (fig. S6).

## Ventral CA1 is required for the influence of latent context on dopamine dynamics

We returned to dopamine recordings to gain further insight into the influence of vCA1 lesions on behavioral strategy. If vCA1 was truly shifting behavior due to a loss of the SI representation, this should be reflected as a loss of SI associated features in NAc dopamine characterized in Fig. 2 (6, 7, 9, 32).

We tested this hypothesis using small, unilateral lesions of excitatory neurons in vCA1 (Fig. 3F). We performed these lesions unilaterally to avoid influence on behavior due to redundancy across hemispheres (fig. S7). Thus, by recording NAc dopamine ipsilaterally to the lesion site, we could monitor the influence of vCA1 on dopamine dynamics, without the confound of altered behavior during our recordings.

Compared to control animals, lesion of vCA1 resulted in NAc dopamine no longer showing features of SI-RPE. This was apparent both by comparing the effect of past trial outcome (Fig. 3G-I), and also using a regression approach (Fig. 3J). Therefore, consistent with our hypothesis, in control animals, NAc dopamine contains strong signatures of SI, while lesions of vCA1 result in an almost complete loss of this influence.

## vCA1 neurons differentiate choice, expected outcome and latent context during task performance

During spatial navigation, ‘place cells’ in the CA1 region of the hippocampus fire when an animal occupies a particular spatial location, and together are proposed to form a ‘map’ of the environment (16–18). This code is proposed to be different for each distinct spatial context encountered, as shown by ‘remapping’: where the neurons that are active in a particular location in one context are not in the equivalent location in another (17–19, 25). This provides a representation of cues and events that is distinct dependent on the context in which they were experienced, allowing for context-specific behavior (34). Because hippocampal representations of behaviorally relevant contexts are also often influenced by non-spatial features such as past rewarding or aversive experiences (1, 3, 19–26), we hypothesized that vCA1 neuronal activity might differentiate the abstract contexts required for performance of the 2-arm bandit task in a similar way to the well documented changes across spatial contexts.

**Fig. 4:**
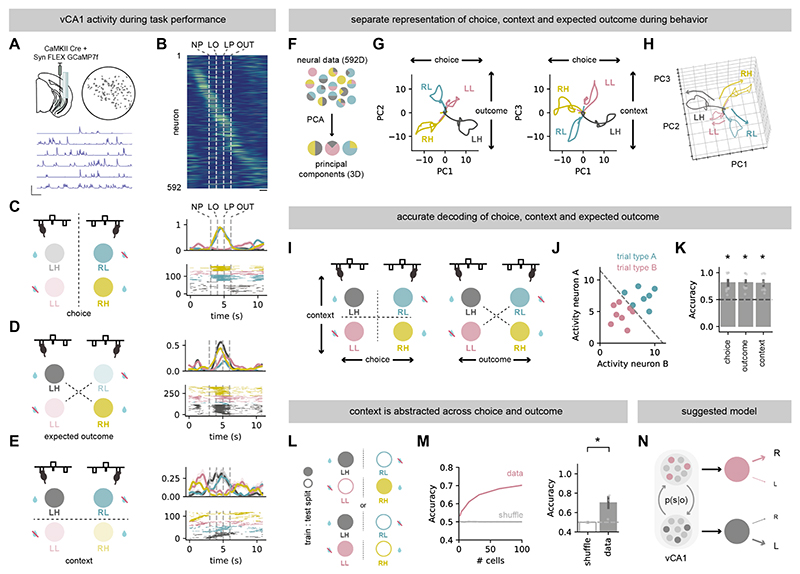
vCA1 maintains a generalized representation of abstract context during task performance. **(A**) Top, schematic of viral injections and GRIN lens placement for Miniscope recordings of vCA1 pyramidal neurons in mice during 2-armed bandit task (left) and example field of view from one recording session (right). Bottom, example activity traces of individual neurons. Scale bar = 2Z, 20 seconds. (**B**) Cross-validated average activity of all recorded neurons, obtained by sorting on ‘even’ trials and plotting ‘odd’ trials. Scale bar = 1s. (**C**) Left, schematic of trials split according to the choice side. Right top, average spike probability of example vCA1 neuron across each trial type. Right, bottom, inferred spikes of the same example neuron. (**D**) Same as (**C**) but for a neuron selective to high reward probability choices across both lever identities. (**E**) Same as (**C**) but for a neuron selective to either choice in a specific context – both left high probability (LH) and right low probability (RL) choices. (**F**) Schematic of principal component analysis of recorded neural activity. (**G**) Top 3 principal components demonstrating separable representations of choice (left or right lever press, PC1), expected outcome (high or low reward probability choice, PC2), or latent context (current block of choice and reward contingencies, PC3). (**H**) 3D representation of three PCs. (**I)** Schematic of different task variable splits used in decoding analysis: choice (RH and RL vs LH and LL), expected outcome (RH and LH vs RL and LL), and latent context (RH and LL vs RL and LH). (**J**) Schematic of SVM analysis. (**K**) Decoding accuracy of animals’ choice, expected outcome, or latent context from activity of simultaneously recorded vCA1 neurons. (**L**) Schematic of a train:test split for decoding of generalized state representation – training performed on one of two conditions in each state (e.g. LH and RH) and then the decoding accuracy tested on the remaining two conditions from the corresponding state (e.g. RL and LL). (**M**) Accuracy of decoding generalized latent context using either neural activity from a pseudo population of between 1 to 100 randomly selected neurons from all sessions from all mice (left) or in individual sessions with over 100 simultaneously recorded neurons (right). (**N**) Schematic of a proposed latent context representation of a 2-armed bandit task by vCA1 pyramidal neurons. Points indicate individual sessions where unique neurons were recorded. Error bars represent s.e.m. across sessions in 3 mice (n = 9). *P < 0.05.

We recorded the activity of neurons in vCA1 using microendoscopic Ca^2+^ imaging in expert mice while they performed the 2-armed bandit task. We imaged a total population of 592 vCA1 pyramidal neurons across 6 sessions from 3 mice while they performed the task (Fig. 4A, B).

In the 2-armed bandit task, in contrast to exploration of dispersed spatial locations, mouse behavior is concentrated around one wall of a small operant chamber. Instead of navigating through a maze, mice progress through different stages of each trial – from nose poke, to lever press to outcome while remaining in approximately the same spatial location. These trials can then be split into 4 types – either right or left choice, and whether that lever is currently associated with a high or low probability of reward delivery (abbreviated here and throughout as right-high: RH (yellow), right-low: RL (cyan), left-high: LH (black), left-low: LL (magenta)). A full representation of the task therefore relies on differentiating three separate organizations of these 4 trial types: i) split by choice (right versus left), ii) split by expected outcome (high versus low probability irrespective of choice), and iii) split by context (right high and left low – ‘context A’, versus right low and left high ‘context B’ – a readout of the contingency of the task).

First we looked for activity of individual neurons that was tuned to specific features of the task. Multiple neurons were active only at specific times across the trial, irrespective of choice or outcome (fig. S8). However, a large proportion of neurons were active only on specific trial types (fig. S8). For example, activity of distinct neurons was separated across choice (~40% of recorded neurons, e.g. Fig. 4C), across the expected outcome irrespective of choice (~20%, e.g. Fig. 4D), but also the contingency of the levers, irrespective or either choice or expected outcome (~30%, e.g. Fig. 4E) – a representation of the latent context. Thus, similar to ‘place cells’ that tile locations and cues and differentiate spatial contexts; there are also vCA1 neurons that tile the different parameter spaces of the 2-arm bandit task, and differentiate the latent contexts utilized to solve the task.

We next asked how the population as a whole represented each of these variables. We first took the average activity of each neuron for each of the 4 trial types, and projected this onto the low dimensional space that captures the greatest variance across the different trial types (Fig. 4F-H). The top three components (PCs) almost perfectly separated trials based on choice (PC1), expected outcome (PC2) or context (PC3). Across these PCs, activity during each trial type was highly separable suggesting rich representations of each trial type and task contingency in hippocampal activity. This same organization was found in individual mice, as well as the overall population (fig. S8).

We hypothesized that this organization should result in robust readout of each task variable. We designed a series of linear decoders to ask to what extent choice, expected outcome and context could be decoded from neural activity from each behavioral session from each mouse, limited to periods before the outcome of each trial (Fig. 4I, J). Using this analysis, consistent with the organization of population activity, choice, expected outcome and context could all be reliably decoded from neuronal data (Fig. 4K). Moreover, by repeating our decoding analysis using small 1 s epochs spread evenly across the trial period, these variables could be decoded stably across each trial and even during the ITI (fig. S8).

## Representations of latent context in vCA1 abstract across choice and outcome

Our results so far suggest that there is a representation of latent context in vCA1. This representation of context could occur via two means. First, individual neurons may represent the interaction between choice and expected outcome. For example, a neuron may fire only on RH trials. Alternatively, vCA1 may contain a more abstract representation of state – where neurons have generalized representations of the latent context, irrespective of the trial type that is currently being performed. Such representations would be like a stable, ‘state’ representation often proposed to be the basis of contextual associative learning (4–10).

Neurons exemplified both scenarios, with individual neurons firing to only one of the 4 trial types (fig. S8), and other neurons that had similar firing patterns across the two distinct trial types within each latent context, but different firing patterns across latent contexts (Fig. 4E, fig. S8). To more quantitively test the presence of this more general representation, we built a separate series of decoders that were trained only on one trial type in each latent context (e.g. RH vs LH, or RH vs RL, Figure 4L). These models were then tested for their ability to accurately decode the other option in the same context (e.g. in an abstracted representation, training on RH trials should be able to correctly predict LL trials in testing). vCA1 activity could decode these abstractions well above chance, both in overall population activity, and on a session-by-session basis (Fig. 4M). Overall, our data are consistent with a model where latent context is represented in vCA1. This stable representation is ideally placed to be used by downstream areas such as prefrontal cortex, orbitofrontal cortex and NAc to define optimal behavior in each context (1–3) (Fig. 4N).

## Discussion

The ability to use subjective experience to guide contextual decision making is fundamental for everyday life, but the neural basis of this ability has remained elusive. In this study we found that the hippocampus - an area strongly associated with spatial contextual representations - supports decision-making utilizing non-spatial, latent contextual representations (35, 36) (Fig. 1-3). Neural activity in vCA1 robustly and stably differentiated two latent contexts formed only from past probabilistic outcomes (Fig. 4) in a manner similar to that utilized to differentiate contexts encountered during spatial navigation (25, 37, 38). Based on the large literature suggesting a key role of the hippocampus in spatial contextual associations (17–19, 26, 37), this suggests a core function for the hippocampus may be in supporting the differentiation of, and inference across, contexts.

Much recent investigation of the basis of hidden state inference has focused on the role of the frontal cortex (FC), including orbitofrontal cortex (OFC) and medial prefrontal cortex (mPFC) (39). Neurons in both rodent and primate FC have strong representations of latent contexts combined with the value associated with different cues and actions within these contexts (3, 40, 41), and activity in these regions is required for signatures of SI to be present in NAc dopamine release (42). It is often presumed that the FC inherits contextual representations from the hippocampus, and uses this information to plan and assign value to cues and actions in each context (1, 2, 39, 43). Indeed, inactivation of ventral hippocampus impairs the representations of latent context in OFC during a similar reversal learning task (44), but the nature of this representation has never been investigated. vCA1 projects strongly to mPFC, and thus indirectly to OFC (44, 45), with specialized connectivity that provides an ideal basis for tight control of FC circuitry by vCA1 (46). We discovered that inhibition of vCA1 impaired both the dopaminergic and behavioral use of latent contexts to perform hidden state inference (Fig. 3) and revealed the neural representations that underlie this key role (Fig. 4). Future work will investigate whether this influence on dopamine signaling and behavior is via vCA1 projections to FC, or via more direct connectivity such as the strong projections from vCA1 to NAc (21, 47–49).

## Materials and Methods

### Animals

6-9 weeks old (adult) male C57BL/6 mice provided by Charles River were used for all experiments. Animals underwent stereotaxic surgery and returned to their home cage for at least 1 week to allow full recovery. Animals were housed in cages of 1 to 4 and kept in a controlled environment under a 12h light/dark cycle with *ad-libitum* access to food and water (unless stated otherwise). All experiments were approved by the UK Home Office as defined by the Animals (Scientific Procedures) Act, and strictly followed University College London ethical guidelines

**Table:** 

Viruses	
AAVl-CaMKII-Cre	Addgene, 105558
AAVl-syn-FLEX-j GCaMP7f-WPRE	Addgene, 104492
AAV5-CAG-dLight1.1	Addgene, 111067
AAV8-hSyn-DIO-mCherry	UNC vector core
AAV5-flex-taCasp3-TEVp	SWC vector core and Addgene, 45580

### Stereotaxic surgery

Stereotaxic surgeries were carried out according to previously described protocols (45, 46, 47, 51). For induction, mice were placed in a red perspex chamber (AN010ASR; VetTech) with 1.75 L/min flow of 4% vaporized isofluorane (in medical oxygen, 99.5% minimum purity). Following induction, fur on the scalp was shaved off using a small trimmer (ChroMini Pro; MOSER), and the animal was secured onto a stereotaxic head frame (Model 902 Dual Small Animal Stereotaxic Instrument; KOPF). Mice were placed on a homeothermic blanket control unit which was maintained between 35 and 37°C throughout the surgery (50-7001; Harvard Apparatus). During induction and throughout the surgery, the induction chamber and the stereotaxic frame were connected to an activated carbon scavenging filter (Cardiff Aldasorber; Shirley Aldred & Co) and an active scavenging unit (Model AN005; VetTech). For the duration of the surgery anaesthesia was maintained at the same flow rate and isofluorane concentration of 1-2%. Ophthalmic ointment (Viscotears® Liquid Gel) was applied to the eyes. The scalp was sterilized with HiBiSCRUB® and the skull was exposed with a single incision along the midline followed by application of a local anesthetic (0.025% Marcaine). After removing the connective tissue with sterile cotton buds, small holes were drilled in the skull at the coordinates of interest using a stainless steel bur (19008-07; Meisinger) attached to a miniature drill (Ideal Micro-Drill®; CellPoint Scientific). Injections were carried out with a Nanoject II (Drummond Scientific) using borosilicate glass pipettes back-filled with mineral oil and front-filled with ~1 μL of the substance to be injected. 120 to 500 nL of virus was injected at a rate of 200 nL/min. Following infusion of the virus, the pipette was left in place for an additional 5 minutes before being slowly retracted. Injection coordinates were as follows (mm relative to bregma):

**Table:** 

Region	ML	RC	DV
Nucleus accumbens (NAc)	± 0.9	+ 1.1	− 4.6
Ventral hippocampus (vH)	± 3.2	− 3.7	− 4.5

After injection, the wound was sutured and sealed. Mice were given a subcutaneous injection of carprofen (0.5 mg/kg) and allowed to recover for a minimum of 30 minutes in a heated chamber before they were returned to their home cage. Animals received carprofen in their drinking water (0.05 mg/mL) for 48 hrs port-surgery.

For photometry experiments, mice were intracranially injected with 200 - 400 nL of *AAV5-CAG-dLight1.1* in NAc. For combined midbrain dopamine photometry and vH genetic lesion experiments, 200 – 400 nL of a 1:1 mix of *AAV1-CaMKII-Cre* and either *AAV8-hSyn-DIO-mCherry* or *AAV5-flex-taCasp3-TEVp* was injected into vH in the same hemisphere as NAc *dLight1.1* injection. Fiber optic cannula (200 μm core diameter, 0.39 NA, 5 mm long; Thorlabs) were implanted unilaterally above NAc following virus injection in the same surgery. To aid cement attachment, the skull was roughened, and two metal screws were inserted into the skull. Fiber implants were secured to the skull by applying two layers of adhesive dental cement (Superbond C&B). The skin was attached to the cured dental cement with Medbond skin glue (Animus).

For bilateral genetic lesion experiments, 200 – 400 nL of a 1:1 mix of *AAV1-CaMKII-Cre* and either *AAV8-hSyn-DIO-mCherry* or *AAV5-flex-taCasp3-TEVp* was bilaterally injected into 4 regions spanning the entirety of vCA1. The wound was sutured (6-0 Coated VICRYL polyglactin 910 suture; ETHICON) and sealed with Medbond skin glue (Animus).

For miniature microscope (UCLA Miniscope, Open Ephys) experiments, surgeries followed previous procedures (52). Briefly, 1 – 2 mm diameter craniotomy was drilled at the vCA1 stereotaxic coordinates and the cortical tissue and corpus callosum fibers were aspirated using a blunt needle connected to a vacuum pump. Sterile saline (BAYER) was applied throughout aspiration to prevent desiccation of the tissue. 400 - 600 nL of a 1:1 mix of AAV1-CaMKII-Cre and pGP-AAV-syn-FLEX-jGCaMP7f-WPRE diluted in 2 parts of sterile saline solution (BAYER) was injected into vCA1. This dilution protocol was used to limit excessive GCaMP7f expression, which could lead to reduced Ca^2+^ variance in the signal, affect cellular processes and reduce cell health (52). To increase the spread of the virus throughout the CA1/subiculum region of the vH, 3 injections of ~165 nL each were delivered at –4.3, –4.5 and –4.7 DV coordinates. A relay gradient refractive index (GRIN) lens (0.6 mm diameter, ~7 mm length, PN 130-000150; or 1 mm diameter, ~4 mm length, PN 130-000143; Inscopix) was implanted either in the same surgery following injection of the viruses or 4 - 6 weeks after the initial surgery fixed to the custom-made base plate (Miniscopeparts) attached to the Miniscope for fluorescence guided implantation. The GRIN lens was inserted at an approximate rate of 0.5 mm/min to a depth between 3.5 – 4.3 mm and secured in place with super glue and further fixed with adhesive dental cement (Superbond C&B). To aid cement attachment, prior to the lens implantation the skull was roughened, and two metal screws were inserted into the skull. The Miniscope base plate was attached with adhesive dental cement (Superbond C&B) above the lens implanted directly to the skull. The base plate was locked to a Miniscope to find the optimal focus in the field of view prior to cementing. A protective cap was attached on top of the base plate to prevent debris build-up.

### Anatomy

#### Histology

Mice were anaesthetized with 0.5 - 1 mL of a mixture of ketamine (100 mg/kg; KetaVet) and xylazine (10 mg/kg; Zoetis) in sterile saline (BAYER). Following confirmation of deep anesthesia, animals were transcardially perfused with ice-cold 4% paraformaldehyde, the brains were dissected and fixed in 4% paraformaldehyde overnight at 4 °C. Brain samples were transferred to phosphate buffered saline (PBS, pH 7.2) after overnight fixation. Coronal brain slices were prepared at 70 µm using a vibratome (Campden Instruments). Slices were then mounted on gelatin-coated Superfrost glass slides with ProLong Gold, ProLong Glass Antifade Mountant with NucBlue (Molecular Probes), or Mowiol mounting medium. Fluorescent images were obtained with a 10x objective using a Zeiss slide scanner Axio Scan.Z1 using a 10x air immersion lens and standard filter sets for excitation/ emission at 365-445/50 nm, 470/40-525/50 nm, 545/25-605/70 nm and 640/30690/50 nm.

#### Immunohistochemistry

Brain slices (70 µm thick) were prepared as above and stained using standard procedures. First, slices were incubated in blocking solution (3% bovine serum albumin, 0.5% triton in PBS) for 1.5 - 3 hours at room temperature (22 – 24 °C) with constant agitation. When using the primary antibody raised in mouse, to eliminate non-specific binding, sections were first incubated overnight at 4 °C with anti-mouse F(ab)’2 Fragment in blocking solution and then washed 3 times for 20 - 40 minutes each in PBS. All slices were incubated overnight at 4 °C in blocking solution containing either 1:1000 anti-GFP (ab13970, Abcam) to reveal dLight1.1-expressing cells in NAc, or 1:500 anti-NeuN (Sigma-Aldrich, ZMS377) and 1:500 anti-GFAP (Dako, GA524) to estimate caspase-induced cell loss in vH. Slices were then washed 3 times for 20 - 40 minutes each wash in PBS before incubation with secondary antibody(s) in blocking solution for 2 - 4 hours at room temperature (Alexa 647-conjugated donkey anti-chicken, AP194SA6, Millipore – to label GFP; Alexa 488- or Alexa 647-conjugated donkey anti-rabbit, A21206 / A31573, Invitrogen – to label GFAP; or Alexa 488- or Alexa 555-conjugated donkey anti-mouse, A21202/ A31570, Invitrogen – to label NeuN). Slides were mounted after a further 3 washes in PBS as above.

### Probabilistic reversal learning task

#### Behavioral setup

We trained animals on a probabilistic reversal learning task (28). Following a minimum of 7 days of recovery after surgery, mice were water-restricted to approximately 85% of their ad-libitum weights. After at least a week of water-restriction and habituation to manual handling by the experimenter, behavioral training for the probabilistic reversal learning task began. All behavioral experiments were performed in 21.59 x 18.08 x 12.7 cm modular operant chambers (MED Associates, ENV-307W). Each chamber was equipped with a stainless-steel grid floor, two stainless steel walls (front and back), and a transparent polycarbonate side-wall, ceiling, and door. The nose port and the stainless steel reward delivery spout were located in the middle of the front wall. Two retractable levers were located either side of the nose port on the front wall (spout placed above the nose port). The behavioral box was also equipped with a house light placed outside of the chamber. Auditory stimuli were presented to animals via a speaker located on the back wall. Experimental events were controlled and recorded using custom scripts in MED-PC IV software.

All training and recording sessions were 1 hour long. Levers out and reward delivery events were separated by a temporal delay drawn from a random distribution from 0.1 to 1 s in 0.1 s intervals. In all stages of the task, a lever press always triggered retraction of the levers. Rewarded trials were signaled by 0.5 s of 5 kHz pure-tone auditory stimulus (C+) and the delivery of 6 µL of reward (10% sucrose in water). Reward omission was signaled by 0.5 s of white-noise (C-). The beginning of each trial was signaled by the illumination of a nose port. All trials were separated by a constant 3 s intertrial interval which began at the end of C+ or C-.

#### Training stages

Prior to data collection, mice went through several stages of training. In the first stage, mice were presented with both levers and had to press either of them to obtain a drop of sucrose solution until they had made over 100 lever presses in a single session. The next stage required mice to learn to nose poke into the central port to initiate presentation of a lever (alternating across trials) that they had to press for reward. Mice were then trained to remain in the nose port for 200 ms: starting with 0 ms, the nose poke duration required for the levers to come out incrementally increased by 10 ms every 10 trials until it reached 200 ms. Following completion of over 100 trials with full 200 ms delay in a single session, mice then progressed to the deterministic reversal learning task. In this training stage, both levers were presented simultaneously but in a given block of trials pressing only one of the two levers would result in a reward. The identity of the rewarded lever reversed after 10 to 32 rewarded trials. After 3 successive sessions of receiving over 100 total rewards and choosing the rewarded lever over 60% of the trials, the mice progressed to the full probabilistic reversal learning task. In the full version of the task, in a given block of trials, one lever was associated with 70% reward probability following a press (high-probability lever) while the opposite lever was rewarded with 10% probability (low-probability lever). The identity of the rewarded lever reversed after 10 to 32 high-probability lever choices. The final choice resulted in an immediate change in contingency. As the outcome of that trial is in the next block, this trial is by definition classed as incorrect. Animals were trained on the full version of the task until they reached the ‘expert’ level with the consistent performance of over 60% high-probability lever choices for 3 consecutive sessions. While training for either the deterministic or probabilistic stage of the task, mice were also habituated to having optic fibers attached to the implanted ferrules or carrying a dummy Miniscope attached to the implanted baseplate until they met the performance criteria of the corresponding stage. Miniscope or photometry recording experiments commenced after mice were fully habituated and met the performance criteria for the final stage of the task.

### Behavioral analysis

To estimate the number of trials taken by mice to switch their choices to a different lever after reward contingencies reversal, we fit the exponential curve to animals’ reversal behavior (proportion of high probability lever presses following the reversal). The fit then allowed us to directly estimate the number of trials taken before animals started choosing the new high probability lever 50% of total lever presses after reversal.

To quantify the influence of the past choice and reward history on animal’s choice on the current trial, we used a logistic regression model (27–29, 53). In this model, the target variable was represented by the probability of the current choice being the right lever press (C(i), 1 if right choice, 0 if left choice). Predictor variable consisted of 3 types of trial history regressors: R(i – j) is the rewarded choice history on trial i – j (1 if rewarded right choice, –1 if rewarded left choice, 0 otherwise), N(i – j) is the unrewarded choice history (1 if unrewarded right choice, –1 if unrewarded left choice, 0 otherwise), C(i – j) is the outcome-independent choice history on trial I – j (1 if right choice, –1 if left choice, 0 otherwise). The encoding model is:

(1) $$log\frac{C(i)}{1-C(i)}=\sum_{j=1}^{n}\beta_{j}^{R}R(i-j)+\sum_{j=1}^{n}\beta_{j}^{N}N(i-j)+\sum_{j=1}^{n}\beta_{j}^{C}C(i-j)+\beta_{0}$$

where $\beta_{j}^{R},\beta_{j}^{N},\beta_{j}^{C}$ are the regression weights of each history predictor, and *β*_0_ is the history-independent constant bias term. While this regressor set is not strictly orthogonal, due to the inclusion of a separate choice predictor, it provides a biologically informed estimate of the contribution of choice and outcome on upcoming choice (29).

To model the animal’s choice given its trial history, the regression coefficients were fit using LogisticRegression function of *scikit-learn* Python library. For this model we used elastic net regularization, method that combines L1 and L2 regularization penalties to minimize the objective function. First, we performed grid search over *C* (inverse of the regularization strength) and λ (L1-ratio) hyperparameter space to find the optimal combination of *C* and λ that explained the most variance when verified with 5-fold cross-validation. The overall total explained variance of the final model *R*^2^ was calculated as an average from 5 cross-validated fits of the model with the best estimated *C* (0.25 ± 0.04) and λ (0.34 ± 0.04) hyperparameters. *C* and λ that provided the best average *R*^2^ score were then used to refit the full data set to obtain estimated regression weights. The logistic regression coefficients were fit separately for each session in each animal. Estimated coefficients represented the extent the different past trial choices and outcomes predicted animals’ current choices. Model *β* coefficients were then used to estimate how much different past trial history predictors influenced animals’ decisions on the current trial.

### Models

We investigated behavioral strategies mice might use when solving the probabilistic reversal learning task by fitting a range of different computational models to their choices. We considered a number of ‘simple’ models such as random choice, win-stay-lose-shift (WSLS) and choice repetition (54) as well as more complex value updating and state inference strategies.

### Simple behavioral models

#### Random choice

Random choice model assumes that mice do not engage with the task and press levers at random with a bias (*b*) for one option over the other. The probabilities of choices *a* and *a’* on trial *t* is:

(2) $$p_{t}(a)=b$$

(3) $$p_{t}\left(a^{\prime }\right)=1-b$$

#### Win-stay / Lose-switch

Noisy WSLS model repeats rewarded actions and switches away from unrewarded actions with probability 1– *ε*/2 and chooses the other option (switching after rewards, staying after losses) with probability *ε*/2 The probability of choosing option *a* is:

(4) $$p_{t+1}(a)=\{\begin{array}{c} 1-\varepsilon /2,\;if\;\left(c_{t}=a\;and\;r_{t}=1\right)OR\left(c_{t}\neq a\;and\;r_{t}=0\right)\\ \;\;\;\;\;\varepsilon /2,\;if\;\left(c_{t}\neq a\;and\;r_{t}=1\right)OR\left(c_{t}=a\;and\;r_{t}=0\right) \end{array}$$

where *c*_*t*_ is the choice at trial *t*, and *r*_*t*_ the reward at trial *t*.

#### Choice kernel

The choice kernel model tries to capture the tendency for mice to repeat their previous actions. Specifically, the agent computes a ‘choice kernel’, *CK*_*t*_(*a*), for each action, which keeps track of how frequently that option was chosen in the past.

The choice kernel updates according to the rule below:

(5) $$CK_{t+1}(a)=CK_{t}(a)+\alpha_{c}\left(c_{t}^{a}-CK_{t}(a)\right)$$

where $c_{t}^{a}=1$ if lever *a* is chosen on trial *t*, otherwise $c_{t}^{a}=0$, and *α*_*c*_ is the choice kernel learning rate. In the choice kernel model each option is chosen according to a softmax function:

(6) $$p_{t+1}(a)=\frac{\exp\left(\beta_{c}\times CK_{t}(a)\right)}{\exp\left(\beta_{c}\times CK_{t}(a)\right)+\exp\left(\beta_{c}\times CK_{t}\left(a^{\prime }\right)\right)}$$

where *β*_*c*_ is the inverse temperature associated with the choice kernel.

### Value updating (Q) models

#### Q-learning

Value updating models are reinforcement learning (RL) models that utilize Q-learning updating rule. In such models on every trial *t* the expected value *Q*_*t*_(*a*) of a chosen action *a* is updated by the reward prediction error (RPE), the difference between the choice outcome *r*_*t*_ and previous expected value, scaled by the learning rate *α*:

(7) $$Q_{t+1}(a)=Q_{t}(a)+\alpha \left(r_{t}-Q_{t}(a)\right)$$

The choice probabilities were estimated based on the action values according to a softmax function:

(8) $$p_{t+1}(a)=\frac{\exp\left(\beta \times Q_{t}(a)\right)}{\exp\left(\beta \times Q_{t}(a)\right)+\exp\left(\beta \times Q_{t}\left(a^{\prime }\right)\right)}$$

where *β* is the inverse temperature.

### Supplemented Q-learning models

Other models from the Q-learning family were augmented alterations of the basic model above.

#### Q-learning with bias

Introducing bias captures an animals’ preference towards one of the levers in the task.

Bias parameter *b* (−1 < *b* < 1) changes the expected value of one of the actions reducing or increasing the probability of choosing that action:

(9) $$p_{t+1}(a)=\frac{\exp\left(\beta \times (Q_{t}(a)+b)\right)}{\exp\left(\beta \times (Q_{t}(a)+b)\right)+\exp\left(\beta \times Q_{t}\left(a^{\prime }\right)\right)}$$

#### Q-learning with choice kernel

Q-learning strategies may also be affected by animals’ tendency to repeat previously selected actions. To incorporate this into the model, we added the choice kernel (eq. 5 and 6) into the softmax decision rule:

(10) $$p_{t+1}(a)=\frac{\exp\left(\beta \times Q_{t}(a)+\beta_{c}\times CK_{t}(a)\right)}{\exp\left(\beta \times Q_{t}(a)+\beta_{c}\times CK_{t}(a)\right)+\exp\left(\beta \times Q_{t}\left(a^{\prime }\right)+\beta_{c}\times CK_{t}\left(a^{\prime }\right)\right)}$$

#### Q-learning with asymmetric updates

Reward and punishment (R/P model) sensitivity augmentation utilizes the same value updating rule while using different learning rates following rewarded (*α*_*r*_) and unrewarded (*α*_*ur*_) outcomes (29, 55):

(11) $$=\{\begin{array}{cc} \alpha_{r}, & \text{ if}\;r_{t}=1\\ \alpha_{ur}, & \;\text{if}\;r_{t}=0 \end{array}$$

#### Q-learning with counterfactual updating

In *counterfactual updating models*, a reward resulting from one choice both increases the expected value of that choice, but also decreases the expected value of the alternative choice (and vice versa following reward omission). In other words the two choices are interdependent. As a result, such models are often described as an approximation of hidden state inference (56), as on each trial the agent infers a change in the value of the unexperienced option (see next section for discussion). In these models, expected values for both actions are updated on every trial: values of the unchosen actions *a*’ are updated according to the counterfactual outcome (1 - *r*_*t*_) from the chosen action (*a*) (5, 12):

(12) $$Q_{t+1}\left(a^{\prime }\right)=Q_{t}\left(a^{\prime }\right)+\alpha \left(\left(1-r_{t}\right)-Q_{t}\left(a^{\prime }\right)\right)$$

We tested four versions of the counterfactual updating models that utilized different sets of learning rates for updating the action values of the chosen and unchosen options following different trial outcomes:

**Table:** 

Model	Chosen action		Unchosen action	
	Rewarded trial	Non-rewarded trial	Rewarded trial	Non-rewarded trial
Same *a;* R/P = False	*a*	*a*	*a*	*a*
Same *a;* R/P = True	*α_r_*	*α_ur_*	*α_r_*	*α_ur_*
Different *a*; R/P = False	*a*	*a*	*a'*	*a'*
Different *a:* R/P = True	*α_r_*	*α_ur_*	*α* *'_r_*	*α* *'_ur_*

#### Q-learning with forgetting

In value updating models with *forgetting*, expected value of the nonchosen action *a*’ was either directly reset over one trial to the average value $\bar{Q_{t}}$ across both actions (Forget reset) or gradually updated towards the average $\bar{Q_{t}}$ according to the forgetting factor *δ* (Forget gradual):

(13) $$Q_{t+1}\left(a^{\prime }\right)=\{\begin{array}{cc} (1-\delta )\times Q_{t}\left(a^{\prime }\right), & \;\text{if}\;Q_{t}\left(a^{\prime }\right)>\bar{Q_{t}}\;\text{and}\;(1-\delta )\times Q_{t}\left(a^{\prime }\right)\geq \bar{Q_{t}}\\ (1+\delta )\times Q_{t}\left(a^{\prime }\right), & \text{if}\;Q_{t}\left(a^{\prime }\right)<\bar{Q_{t}}\;\text{and}(1+\delta )\times Q_{t}\left(a^{\prime }\right)\leq \bar{Q_{t}}\\ \bar{Q_{t}}, & \;\text{if}\;Q_{t}\left(a^{\prime }\right)>\bar{Q_{t}}\;\text{and}\;(1-\delta )\times Q_{t}\left(a^{\prime }\right)\leq \bar{Q_{t}}\\ \bar{Q_{t}}, & \text{if}\;Q_{t}\left(a^{\prime }\right)<\bar{Q_{t}}\;\text{and}\;(1-\delta )\times Q_{t}\left(a^{\prime }\right)\geq \bar{Q_{t}} \end{array}$$

#### Q-learning with dynamic value updating

*Dynamic value updating models* are based on the basic RL strategy that utilizes the Pearce-Hall rule (57, 58). These models contain an associability parameter that modulates the learning rate as a function of the absolute magnitude of past RPEs. The *κ* parameter modulates the action value updating and is equivalent to the learning rate parameter in the basic Q-learning models. On the first trial, *a*_*t*_ is a free parameter. The *γ* parameter controls the temporal dynamics of associability over time:

(14) $$Q_{t+1}(a)=Q_{t}(a)+\kappa \times \alpha_{t}\left(r_{t}-Q_{t}(a)\right)$$

(15) $$\alpha_{t}=\alpha_{t-1}(1-\gamma )+\gamma \times \left|{\text{RPE}}_{t-1}\right|$$

where we noted that |RPE| <= 1 and therefore *a* remains appropriately bounded. Like other value updating models, dynamic value updating models could also be modified to include bias, perseverance, and R/P as described above. R/P is enabled by having different *κ* parameters for rewarded and unrewarded trial outcomes:

(16) $$\kappa =\{\begin{array}{c} \kappa_{r},\;\text{if}\;r_{t}=1\\ \kappa_{ur},\text{ if}\;r_{t}=0 \end{array}$$

### State inference (SI) models

#### State inference

State inference models use Bayesian inference and assume that on each trial mice chose their actions based on their belief *b*_*t*_(*s*) = *p*(*s*_*t*_|*o*^*t*-1^) about the underlying state of the task *s*_*t*_ given the history of observed outcomes *o*^*t*-1^. In this formulation, action-reward pairs on a given trial are treated as simple observations: *o*_*t*_ = {*a*_*t*_,*r*_*t*_}.

The belief variable takes on a role similar to the Q value in the standard Q-learning models above, becoming a function of the past observations and the parameters (12):

(17) $$p\left(s_{t+1}|o^{t}\right)=p\left(s_{t+1}|s_{t}\right)\times \frac{p\left(o_{t}|s_{t}\right)p\left(s_{t}|o^{t-1}\right)}{p\left(o_{t}|s_{t}\right)p\left(s_{t}|o^{t-1}\right)+p\left(o_{t}|s_{t}^{\prime }\right)p\left(s_{t}^{\prime }|o^{t-1}\right)}$$

where *p*(*o*_*t*_|*s*_*t*_) the probability of an observation *o*_*t*_ at trial *t*, is defined by its ‘compatibility’ with the state *s*_*t*_, using parameter *c*:

(18) $$p\left(o_{t}|s_{t}\right)=\frac{1}{2}+\frac{1}{2}\times \{\begin{array}{cc} +c, & \text{if }a_{t}=s_{t}\;\text{and}\;r_{t}=1\\ -c, & \text{if }a_{t}\neq s_{t}\;\text{and}\;r_{t}=1\\ -c, & \text{if }a_{t}=s_{t}\;\text{and}\;r_{t}=0\\ +c, & \text{if }a_{t}\neq s_{t}\;\text{and}\;r_{t}=0 \end{array}$$

and the transition probability of the state *p*(*s*_*t*+1_|*s*_*t*_) is parameterized by a single parameter *γ* - the probability of staying in a state:

(19) $$p(S_{t+1}|S_{t})=\left[\begin{array}{cc} 0.5+0.5\gamma & 0.5-0.5\gamma \\ 0.5-0.5\gamma & 0.5+0.5\gamma \end{array}\right]$$

Finally, the current belief *b*_*t*_(*s*) about the state of the task based on past observations is mapped into action probabilities via a softmax function as in Q-learning models. Note that steepness of the sigmoid is fixed to 10 as this trades off with the state estimate (12).

(20) $$p_{t+1}(a)=\frac{\exp\left(10\times b_{t}(s)\right)}{\exp\left(10\times b_{t}(s)\right)+\exp\left(10\times b_{t}\left(s^{\prime }\right)\right)}$$

#### Supplemented state inference models

Similar to Q-learning agents, SI models were also supplemented with choice kernel and bias terms. In addition, similar to the R/P augment in the Q-learning models, in the state inference model with reward and punishment sensitivity, different probability parameter *d* may be used following reward omission:

(21) $$p\left(o_{t}|s_{t}\right)=\frac{1}{2}+\frac{1}{2}\times \{\begin{array}{cc} +c, & \text{if }a_{t}=s_{t}\;\text{and}\;r_{t}=1\\ -c, & \text{if }a_{t}\neq s_{t}\;\text{and}\;r_{t}=1\\ -d, & \text{if }a_{t}=s_{t}\;\text{and}\;r_{t}=0\\ +d, & \text{if }a_{t}\neq s_{t}\;\text{and}\;r_{t}=0 \end{array}$$

In most fits to animals’ behavior the reward omission update parameter *d* was estimated to be very close to 0, therefore we also tested a model fixing parameter *d* at 0. Thus, following reward omission *p*(*o*_*t*_|*s*_*t*_) is 0.5 for both actions.

### Model fitting

To estimate the values of the parameters that best describe the behavioral data, we used likelihood maximization approach to model fitting (54). For this, for each behavioral session we estimated the probability of individual choices based on a given model (*m*), parameters of the model (Θ_*m*_) and choice and outcome history in that session. We then summed the logs of choice probabilities that corresponded to animals’ choices on every given trial. The python function *scipy.optimize.minimize* was used to find the set of parameter values that minimized the negative log of the likelihood of the data (*LL*) given the model parameters *p*(*d*_1:*T*_|Θ_*m*_, *m*):

(22) $$LL=\log p\left(d_{1:T}|{\text{Θ}}_{m},m\right)=\sum_{t=1}^{T}\log p\left(c_{t}|d_{1:t-1},{\text{Θ}}_{m},m\right)$$

To avoid finding the local minima in the minimization procedure, we repeated model fitting procedure 50 times using randomly selected initial values from defined bounds for each parameter, and recorded the best fitting log likelihood for each run. The best fitting parameters were selected from the run with the highest log-likelihood value.

To determine which model provided the most parsimonious fit to the data we compared different model fits to each individual session using Bayesian Information Criterion (BIC). BIC has an explicit penalty for the number of free parameters (*k*_*m*_) in the model *m* and thus controls for overfitting:

(23) $$BIC=-2\log\hat{LL}+k_{m}\log(N)$$

where $\hat{LL}$ is the log-likelihood value at the best fitting parameters, and *N* is the number of trials in a session. To compare the model fits for each animal, we then computed the differences between the BIC scores of each model fit to individual behavioral sessions with the BIC score of the most parsimonious model of the same session (ΔBIC). In fig. S3 we additionally calculate an alternate ΔBIC where all model fits to each session are compared explicitly to the SI model used in the main figures.

### Determination of example models for main comparison

According to the BIC analysis, all behavioral sessions from all mice were best described by SI models. Specifically, the most parsimonious SI models had reward and punishment sensitivity (R/P) and used only rewarded trials for *p*(*o*_*t*_|*s*_*t*_) updates (*d* = 0); while some also had choice bias. This means that the ‘best’ SI model uses *p*(*o*_*t*_|*s*_*t*_) *p*(*s*_*t*+1_|*s*_*t*_) and *p*(*s*_*t*_|*o*^*t-*1^) as part of its estimation of the current state on rewarded trials, but on unrewarded trials *p*(*o*_*t*_|*s*_*t*_) = 0.5 and therefore only *p*(*s*_*t*+1_|*s*_*t*_) and *p*(*s*_*t*_|*o*^*t*-1^) influence the belief update. This strategy appears to be common during performance of probabilistic behavior in mice (60), and allows a tolerance for high reward omission rates on the correct option, enabling stable performance at relatively low reward probabilities. Among the Q model group, the most parsimonious models either had only supplemented bias parameters, or had asymmetric learning rates for rewarded and unrewarded outcomes (R/P) as well as choice bias.

Therefore, for our comparison of the SI and Q models in the main figures, we focused on the versions of Q and SI models that included the same augmentations – R/P and choice bias.

Importantly, we focused on Q-learning without counterfactual updating in the main figures, as – because this model is commonly seen as an approximation of SI (56) – we wanted to avoid confounds due to the presence of inference in our main comparisons. However, for completeness we also include comparisons of both behavior (fig. S2) and dopamine (fig. S5) predictions from supplemented Q-learning models (including counterfactual updating, dynamic learning rates and forgetting). In these comparisons we also compared exemplar models with the same augmentations for consistency (R/P and choice bias). These specific models are highlighted in fig. S2 and S3, and details of parameters for each model are outlined in Table S3. In all cases, predictions from SI models were most consistent with the data. The ability of each model to describe data in each part of the study are summarized in fig. S9. To assess changes in behavior in caspase lesion/sham animals (Fig. 3C-E), mouse behavior was fit as above from sessions obtained at baseline before lesion. The effects of the lesion or sham were then assessed using these model parameters to investigate how model predictions of trial-by-trial behavior were altered by the manipulation.

### Model simulations

To simulate the probabilistic reversal learning behavior, we ran the models with the parameter sets obtained from the model fits to individual mouse sessions. Each set of parameters was used for 3 simulation runs, and a simulation run comprised of 300 trials. To obtain trial-by-trial choice probabilities from different strategies (Fig. 1H and fig. S1, S2) we used sets of average parameters from model fits to update model predictions based on animals’ choices and outcomes on a trial-by-trial basis (9,30).

### Model verification

To check how reliably we can conclude that the best model from the fitting procedure was more likely to have generated the data compared to other models that were tested we performed model recovery (54). We approached this in 3 stages, directly comparing the Q and SI models used in the main figures (fig. S1), comparing the exemplar models of each class (fig. S2), and finally comparing all models with all supplements (fig. S3). For each of these analyses, we used simulated data from all models and fit that data with all models. From this we quantified the proportion of the simulated data generated by one model that was best fit other models *p*(fit model|simulated model), summarized in a confusion matrix. If 100 % of the simulations were best fit by the same models that produced the simulated data, the confusion matrix would be the identity matrix. We also computed the inversion matrix that quantified the probability the model generated the data given that it provided the best fit *p*(simulated model|fit model). From the inversion matrix we can estimate the confidence with which we can draw conclusions about the behavioral strategies based on the best fitting models (how likely the same model is to have generated the data). As evidenced from these figures (and in particular fig. S2 and S3), while there was confusion within class, for example between different implementations of Q-learning; there was very little confusion across strategies (see fig. S1). Therefore the model fitting approach allows investigation of Q vs SI strategies in mouse behavior.

### Photometry

#### Recording setup

To measure dopamine release, we recorded dLight1.1 fluorescence using a custom-built fiber photometry acquisition as described previously (46, 51). Briefly, to record dLight-dependent fluorescence we used blue 470 nm LED, while to control for dopamine-independent fluctuations in recorded fluorescence (e.g. due to movement) we used violet 405 nm LED. LEDs were controlled via a custom script written in LabView (National Instruments). To enable synchronization with the behavioral task, the recording was initiated by a TTL pulse from the MED-PC program at the start of the behavioral session. To ensure the separation of the blue and the violet channels, the light amplitudes were modulated sinusoidally with two different frequencies (500 Hz and 210 Hz, respectively). For excitation, light from both LEDs passed through corresponding excitation filters (470 nm and 405 nm) before being combined into a single beam by a dichroic mirror. The excitation light was then passed through a beam splitter to allow for simultaneous recordings in two animals. The excitation beams were then reflected off a dichroic mirror, collimated and launched into a fiber patch cord (200 µm core and 0.22 NA). The patch cord was connected to the ferrule of the implanted optical cannula on the animal’s head via an interconnect. The emission signal was passed through the same patch cord and collimator, and filtered through an emission filter (transmission above 505 nm). It then passed through a dichroic mirror and focused onto a femtowatt photoreceiver (Newport) sampling at 10 kHz. Each of the two modulated signals generated by the two LEDs was recovered using standard demodulation techniques implemented by a custom Labview script. dLight and control autofluorescence signals were then downsampled to 500 Hz before being exported for further analysis.

#### Photometry data processing

Photometry data were analyzed with custom-written Python scripts. First, to reduce the noise, a lowpass filter was used on both dLight and control signal. To correct for photobleaching, a 4th order polynomial fit was subtracted from each trace. The fluorescent signal obtained after stimulation with control 405 nm LED was used to correct for dopamine-independent changes in fluorescence such as due to movement. Movement artifacts were estimated by a least-squares linear fit of the control signal from 405 LED excitation to the dLight fluorescence. The estimated movement signal was then subtracted from the dLight signal to obtain the movement-corrected signal corresponding to dopamine release. Signals were then z-score normalized. For photometry experiments with chronic taCasp3 hippocampal inactivations, we excluded data from mice where dLight signals were not observable (2 mice from mCherry control group), or where mice had misplacement of caspase injections inferred from the immunohistochemistry labelling (2 mice from taCasp3 group).

#### Photometry data analysis

Dopamine signals were analyzed in two complementary ways. First, z-scored signals were aligned to C+ or C- and baselined to the mean signal from 1 s preceding the event. The event summary was obtained by calculating mean of the baselined signal in the first 4 seconds of the event. Selection of a wide time window for the event summary enabled us to capture most of the event-associated signal in an unbiased way irrespective of the temporal variability across animals. These events were then sorted according to past choice and outcome history (same or opposite choice, rewarded or non rewarded outcome), and compared to RPE calculated from simulations of agents utilizing Q or SI strategies. For Q estimates, RPE on a particular trial was the outcome minus the estimated value for that choice. For SI estimates, RPE was the outcome minus the reward estimated from the reward probability matrix. We compared model estimates qualitatively across pairs of choices and outcomes (fig. S2), but also investigated this more quantitively using a regression approach to predict dLight signal across each of the 8 trial types using either SI and Q RPE as predictors. Second, we used 2-fold cross validated ridge regression to express dLight fluorescence as a sum of responses related to outcome, past outcome, choice and past choice, as well as estimates of Q-RPE and SI-RPE from our model fits. For this analysis, photometry traces were aligned across trials by linearly time-warping the signal at the intervals between different fixed trial events and resampling the signal at a fixed rate. We only included data within 6 trials of a switch in contingency, due to the increased number of incongruent trials allow better discrimination of the two RPE predictors. Behavioral predictors (outcome, past outcome, choice and past choice) were binary variables centered at 0 (i.e. outcomes were coded as 0.5 for rewarded and -0.5 for non-rewarded), while latent variables (SI- and Q-RPE) were continuous estimates from model fits. For each time point we calculated the coefficient of partial determination (CPD) for each predictor, i.e., what percentage of the variance of the dLight activity at that time-point was explained by the full regression analysis that was not explained by the regression analysis if that predictor was removed. To compliment this we also performed single predictor regressions where we calculated the variance that could be explained by only one predictor. For both of these metrics we compared the contribution of SI- and Q-RPE to model fits, and the influence of vCA1 lesions.

### Miniscope

#### Recording setup

Calcium imaging was acquired using Miniscope V4 – a head-mounted microscope (OpenEphys) controlled via Miniscope-DAQ-QT-Software. A blue LED was used for excitation (~470 nm spectral peak) with power adjusted to approximately match the mean brightness of the image across animals. Fluorescence was passed through an emission filter (bandpass filter, 525/50 nm) and collected by a CMOS imaging sensor. Before the start of the recording, the Miniscope was attached to the base plate and its focal plane was adjusted. Afterwards, the mouse with the Miniscope attached was placed in a behavioral chamber (MED Associates, ENV-307W) for 3–5 minutes before the recording session started. Miniscope was connected to a laptop via a flexible coaxial cable and an off-board data acquisition (DAQ) board and the calcium imaging data was acquired at 30 Hz using Miniscope-DAQ-QT-Software (https://github.com/Aharoni-Lab/Miniscope-DAQ-QT-Software#miniscope-daq-qt-software).

#### Miniscope data processing

Minian software was used for all pre-processing stages and subsequent fluorescence signal extraction (61). To improve the computational performance of the processing pipeline, the videos were first cropped to a rectangle containing the imaged cells, the video width and height was down sampled by a factor of 2, and the framerate was down sampled by a factor of 2. Following the correction of the background fluorescence and median filtering for sensor noise removal, the video was motion-corrected and seeds for estimation of cells’ spatial footprints were initialized. This set of seeds was then used for cell and signal detection using a constrained non-negative matrix factorization (CNMF) algorithm (62). Following the refinement of the spatial footprints and denoising of the temporal traces of each cell, the CNMF algorithm produced background-subtracted calcium fluorescence values and deconvolved the calcium trace into estimated ‘spikes’ that corresponded to a scaled probability of neural activity. The results were then visually inspected and non-cell like shapes and traces were excluded from the output. Deconvolved calcium traces were subsequently aligned across trials by time-warping as described for photometry above.

#### Selectivity index analysis

Trial type selectivity of individual neurons was computed as:

(24) $$SI=\frac{f_{+}-f_{-}}{f_{+}+f_{-}}$$

where *f*_+_ and *f*_-_ are the average activity of the neuron in the period from 1s before trial initiation up to the C+ or C- delivery on different trial types (right vs left, high or low reward probability, state A vs state B choices) (63). To assess the statistical significance of selectivity indices (SIs) of individual neurons, we compared their SI values to those derived from 1000 shuffled datasets, where the labels of trial types were randomly reassigned.

#### Neuronal trajectory analysis

The neural population activity trajectories were obtained by projecting the average population activity for each trial type into the low dimensional space that captured most variance between trial types. Every trial in the task belonged to one of four conditions defined by the combination of animal’s choice and reward contingency associated with the chosen lever in a current block of trials: left-high (LH), right-high (RH), left-low (LL), and right-low (RL). First, to evaluate the component of activity that was not selective to different trial types, we calculated the average activity for each neuron across all trial types. We then subtracted the non-selective activity for each neuron from that neurons average activity for each individual trial type, baselined to isolate within-trial variation, and concatenated across trial types to generate a data matrix representing how activity for each neuron deviated from its cross-trial-type average in each trial type (64). We performed PCA on this matrix to find the space that captured the most cross-trial-type variance and then projected the average population activity trajectory for each trial type into this space.

#### Population decoding analysis

The decoding analysis was used to predict different trial types based on mean spiking probability of simultaneously recorded neurons. For this, average neural responses associated with different task variables we estimated as a mean spiking probability of individual neurons from 1s before the trial initiation up until C+ or C-. Based on different combinations of animal’s choices and associated reward probabilities outlined above, each trial could be classified based on animal’s choice (lever identity – LH and LL vs RH and RL), expected outcome (reward probability associated with the chosen lever – LH and RH vs LL and RL) or task state (a set of reward contingencies associated with either choice in a given block of trials – LH and RL vs LL and RH). To balance the number of different trial types, for each neuron each class trial pool was randomly sampled 250 times with replacement. Unless states otherwise, the decoding analysis was performed on activity from simultaneously recorded neurons from a single behavioral session.

For decoding, we used a support vector machines (SVM) classifier with a linear kernel implemented through LinearSVC function from *scikit-learn* Python library. For cross-validation, data were randomly divided into two non-overlapping groups of trials, used for training and testing the classifiers (75/25% split). The decoder was trained to discriminate between population responses corresponding to two sets of trial variables representing either animal’s choice, expected outcome, or task state. The regularization hyperparameter C was optimized using GridSearchCV *scikit-learn* function with 5-fold cross-validation. The decoding performance was then tested using a held-out test set. This procedure was repeated at least 100 times for each classifier with random train/test subdivisions and the decoding accuracy was computed as the average result across repetitions. To assess the statistical significance of the decoding accuracy, we repeated the same procedure described above on a dataset with shuffled trial labels.

For cumulative decoding plots, we generated neural pseudo-populations from subsets of neurons sampled across multiple animals and/or recording FOVs. Each trial condition was sampled 250 times and activity of individual cells within that condition shuffled. The decoding analysis was performed as for single session models outlined above.

#### Generalization decoding analysis

To quantify the degree of generalized representation of the trial variables, we used cross-condition generalization performance (65). In generalization decoding analysis training and testing sets were created by splitting trials according to their trial labels, so the decoder was trained to discriminate trial categories according to half of the labels and then the discrimination generalization was assessed on the data from different conditions not used in training. For example, to test generalized encoding of choice, the decoder was trained to discriminate RH and LH trials and its performance was tested on discrimination of RL and LL trials, respectively.

### Statistical analysis

All statistics were calculated using the Python packages *scipy, pingouin* and *statsmodels*, and *lme4* R package implemented in Python through rpy2. Summary data are reported as mean ± s.e.m. (standard error of the mean). Unless otherwise stated, statistical tests were performed comparing data from individual behavioral sessions, including mouse identity as a random effect to maintain the dependence between sessions from individual mice. As a result, p values are estimated using the Satterthwaite approximation. Normality of data distributions was determined by visual inspection of the data points. Test statistics are detailed in the supplementary statistics table. Threshold for statistical significance was defined as 0.05. Animals were randomly assigned to a virus cohort (e.g. sham versus lesion), and as far as possible, littermates testing each variable of interest were present in each cohort to control for experiment-to-experiment variability. The experimenter was not blinded to each mouse’s assignment. No power analysis was run to determine sample size a priori. The sample sizes chosen are similar to those used in previous publications. Throughout the figures the * symbol represents *p* < 0.05.

## Supplementary Material

MDAR Reproducibility Checklist

Supplementary Materials

## Data Availability

All data are available in the main text or supplementary materials and are deposited at Zenodo (50).
