# Supplementary material for: Hidden state inference requires abstract contextual representations in ventral hippocampus
Source: Science. Author manuscript; Available in PMC 2025 Nov 14. (PMC7618349; doi:10.1126/science.adq5874)
Supplement: Supplementary Materials [file EMS210473-supplement-Supplementary_Materials.pdf]

Science
AAAS

Supplementary Materials for

Hidden state inference requires abstract contextual representations in the ventral hippocampus

Karyna Mishchanchuk *et al.*

Corresponding author: Andrew F. MacAskill, a.macaskill@ucl.ac.uk

Science **386**, 926 (2024)
DOI: 10.1126/science.adq5874

The PDF file includes:

Materials and Methods
Figs. S1 to S9
Tables S1 to S3
References

Other Supplementary Material for this manuscript includes the following:

MDAR Reproducibility Checklist

Materials and Methods

Animals

6-9 weeks old (adult) male C57BL/6 mice provided by Charles River were used for all experiments. Animals underwent stereotaxic surgery and returned to their home cage for at least 1 week to allow full recovery. Animals were housed in cages of 1 to 4 and kept in a controlled environment under a 12h light/dark cycle with *ad-libitum* access to food and water (unless stated otherwise). All experiments were approved by the UK Home Office as defined by the Animals (Scientific Procedures) Act, and strictly followed University College London ethical guidelines

Viruses

AAV1-CaMKII-Cre	Addgene, 105558
AAV1-syn-FLEX-jGCaMP7f-WPRE	Addgene, 104492
AAV5-CAG-dLight1.1	Addgene, 111067
AAV8-hSyn-DIO-mCherry	UNC vector core
AAV5-flex-taCasp3-TEVp	SWC vector core and Addgene, 45580

Stereotaxic surgery

Stereotaxic surgeries were carried out according to previously described protocols (*45*, *46*, *47*, *51*). For induction, mice were placed in a red perspex chamber (AN010ASR; VetTech) with 1.75 L/min flow of 4% vaporized isofluorane (in medical oxygen, 99.5% minimum purity). Following induction, fur on the scalp was shaved off using a small trimmer (ChroMini Pro; MOSER), and the animal was secured onto a stereotaxic head frame (Model 902 Dual Small Animal Stereotaxic Instrument; KOPF). Mice were placed on a homeothermic blanket control unit which was maintained between 35 and 37°C throughout the surgery (50-7001; Harvard Apparatus). During induction and throughout the surgery, the induction chamber and the stereotaxic frame were connected to an activated carbon scavenging filter (Cardiff Aldasorber; Shirley Aldred & Co) and an active scavenging unit (Model AN005; VetTech). For the duration of the surgery anesthesia was maintained at the same flow rate and isofluorane concentration of 1-2%. Ophthalmic ointment (Viscotears® Liquid Gel) was applied to the eyes. The scalp was sterilized with HiBiSCRUB® and the skull was exposed with a single incision along the midline followed by application of a local anesthetic (0.025% Marcaine). After removing the connective tissue with sterile cotton buds, small holes were drilled in the skull at the coordinates of interest using a stainless steel bur (19008-07; Meisinger) attached to a miniature drill (Ideal Micro-Drill®; CellPoint Scientific). Injections

were carried out with a Nanoject II (Drummond Scientific) using borosilicate glass pipettes back-filled with mineral oil and front-filled with ~1 μL of the substance to be injected. 120 to 500 nL of virus was injected at a rate of 200 nL/min. Following infusion of the virus, the pipette was left in place for an additional 5 minutes before being slowly retracted. Injection coordinates were as follows (mm relative to bregma):

**Region**	**ML**	**RC**	**DV**
Nucleus accumbens (NAc)	± 0.9	+ 1.1	− 4.6
Ventral hippocampus (vH)	± 3.2	− 3.7	− 4.5

After injection, the wound was sutured and sealed. Mice were given a subcutaneous injection of carprofen (0.5 mg/kg) and allowed to recover for a minimum of 30 minutes in a heated chamber before they were returned to their home cage. Animals received carprofen in their drinking water (0.05 mg/mL) for 48 hrs port-surgery.

For photometry experiments, mice were intracranially injected with 200 - 400 nL of *AAV5-CAG-dLight1.1* in NAc. For combined midbrain dopamine photometry and vH genetic lesion experiments, 200 – 400 nL of a 1:1 mix of *AAV1-CaMKII-Cre* and either *AAV8-hSyn-DIO-mCherry* or *AAV5-flex-taCasp3-TEVp* was injected into vH in the same hemisphere as NAc *dLight1.1* injection. Fiber optic cannula (200 μm core diameter, 0.39 NA, 5 mm long; Thorlabs) were implanted unilaterally above NAc following virus injection in the same surgery. To aid cement attachment, the skull was roughened, and two metal screws were inserted into the skull. Fiber implants were secured to the skull by applying two layers of adhesive dental cement (Superbond C&B). The skin was attached to the cured dental cement with Medbond skin glue (Animus).

For bilateral genetic lesion experiments, 200 – 400 nL of a 1:1 mix of *AAV1-CaMKII-Cre* and either *AAV8-hSyn-DIO-mCherry* or *AAV5-flex-taCasp3-TEVp* was bilaterally injected into 4 regions spanning the entirety of vCA1. The wound was sutured (6-0 Coated VICRYL polyglactin 910 suture; ETHICON) and sealed with Medbond skin glue (Animus).

For miniature microscope (UCLA Miniscope, Open Ephys) experiments, surgeries followed previous procedures (*52*). Briefly, 1 – 2 mm diameter craniotomy was drilled at the vCA1 stereotaxic coordinates and the cortical tissue and corpus callosum fibers were aspirated using a blunt needle connected to a vacuum pump. Sterile saline (BAYER) was applied throughout aspiration to prevent desiccation of the tissue. 400 - 600 nL of a 1:1 mix of AAV1-CaMKII-Cre and pGP-AAV-syn-FLEX-jGCaMP7f-WPRE diluted in 2 parts of sterile saline solution (BAYER) was injected into vCA1. This dilution protocol was used to limit excessive GCaMP7f expression, which could lead to reduced Ca^{2+} variance in the signal, affect cellular processes and reduce cell health (*52*). To increase the spread of the virus throughout the CA1/subiculum region of the vH, 3 injections of ~165 nL each were delivered at –4.3, –4.5 and –4.7 DV coordinates. A relay gradient

index (GRIN) lens (0.6 mm diameter, ~7 mm length, PN 130-000150; or 1 mm diameter, ~4 mm length, PN 130-000143; Inscopix) was implanted either in the same surgery following injection of the viruses or 4 - 6 weeks after the initial surgery fixed to the custom-made base plate (Miniscopeparts) attached to the Miniscope for fluorescence guided implantation. The GRIN lens was inserted at an approximate rate of 0.5 mm/min to a depth between 3.5 – 4.3 mm and secured in place with super glue and further fixed with adhesive dental cement (Superbond C&B). To aid cement attachment, prior to the lens implantation the skull was roughened, and two metal screws were inserted into the skull. The Miniscope base plate was attached with adhesive dental cement (Superbond C&B) above the lens implanted directly to the skull. The base plate was locked to a Miniscope to find the optimal focus in the field of view prior to cementing. A protective cap was attached on top of the base plate to prevent debris build-up.

Anatomy

Histology. Mice were anaesthetized with 0.5 - 1 mL of a mixture of ketamine (100 mg/kg; KetaVet) and xylazine (10 mg/kg; Zoetis) in sterile saline (BAYER). Following confirmation of deep anesthesia, animals were transcardially perfused with ice-cold 4% paraformaldehyde, the brains were dissected and fixed in 4% paraformaldehyde overnight at 4 °C. Brain samples were transferred to phosphate buffered saline (PBS, pH 7.2) after overnight fixation. Coronal brain slices were prepared at 70 µm using a vibratome (Campden Instruments). Slices were then mounted on gelatin-coated Superfrost glass slides with ProLong Gold, ProLong Glass Antifade Mountant with NucBlue (Molecular Probes), or Mowiol mounting medium. Fluorescent images were obtained with a 10x objective using a Zeiss slide scanner Axio Scan.Z1 using a 10x air immersion lens and standard filter sets for excitation/ emission at 365-445/50 nm, 470/40-525/50 nm, 545/25-605/70 nm and 640/30690/50 nm.

Immunohistochemistry. Brain slices (70 µm thick) were prepared as above and stained using standard procedures. First, slices were incubated in blocking solution (3% bovine serum albumin, 0.5% triton in PBS) for 1.5 - 3 hours at room temperature (22 – 24 °C) with constant agitation. When using the primary antibody raised in mouse, to eliminate non-specific binding, sections were first incubated overnight at 4 °C with anti-mouse F(ab)'2 Fragment in blocking solution and then washed 3 times for 20 - 40 minutes each in PBS. All slices were incubated overnight at 4 °C in blocking solution containing either 1:1000 anti-GFP (ab13970, Abcam) to reveal dLight1.1-expressing cells in NAc, or 1:500 anti-NeuN (Sigma-Aldrich, ZMS377) and 1:500 anti-GFAP (Dako, GA524) to estimate caspase-induced cell loss in vH. Slices were then washed 3 times for 20 - 40 minutes each wash in PBS before incubation with secondary antibody(s) in blocking solution for 2 - 4 hours at room temperature (Alexa 647-conjugated donkey anti-chicken, AP194SA6, Millipore – to label GFP; Alexa 488- or Alexa 647-conjugated donkey anti-rabbit, A21206 / A31573, Invitrogen – to label GFAP; or Alexa 488- or Alexa 555-conjugated donkey anti-mouse, A21202/ A31570, Invitrogen – to label NeuN). Slides were mounted after a further 3 washes in PBS as above.

Probabilistic reversal learning task

Behavioral setup. We trained animals on a probabilistic reversal learning task (*28*). Following a minimum of 7 days of recovery after surgery, mice were water-restricted to approximately 85% of their ad-libitum weights. After at least a week of water-restriction and habituation to manual handling by the experimenter, behavioral training for the probabilistic reversal learning task began. All behavioral experiments were performed in 21.59 x 18.08 x 12.7 cm modular operant chambers (MED Associates, ENV-307W). Each chamber was equipped with a stainless-steel grid floor, two stainless steel walls (front and back), and a transparent polycarbonate side-wall, ceiling, and door. The nose port and the stainless steel reward delivery spout were located in the middle of the front wall. Two retractable levers were located either side of the nose port on the front wall (spout placed above the nose port). The behavioral box was also equipped with a house light placed outside of the chamber. Auditory stimuli were presented to animals via a speaker located on the back wall. Experimental events were controlled and recorded using custom scripts in MED-PC IV software.

All training and recording sessions were 1 hour long. Levers out and reward delivery events were separated by a temporal delay drawn from a random distribution from 0.1 to 1 s in 0.1 s intervals. In all stages of the task, a lever press always triggered retraction of the levers. Rewarded trials were signaled by 0.5 s of 5 kHz pure-tone auditory stimulus (C+) and the delivery of 6 µL of reward (10% sucrose in water). Reward omission was signaled by 0.5 s of white-noise (C-). The beginning of each trial was signaled by the illumination of a nose port. All trials were separated by a constant 3 s intertrial interval which began at the end of C+ or C-.

Training stages. Prior to data collection, mice went through several stages of training. In the first stage, mice were presented with both levers and had to press either of them to obtain a drop of sucrose solution until they had made over 100 lever presses in a single session. The next stage required mice to learn to nose poke into the central port to initiate presentation of a lever (alternating across trials) that they had to press for reward. Mice were then trained to remain in the nose port for 200 ms: starting with 0 ms, the nose poke duration required for the levers to come out incrementally increased by 10 ms every 10 trials until it reached 200 ms. Following completion of over 100 trials with full 200 ms delay in a single session, mice then progressed to the deterministic reversal learning task. In this training stage, both levers were presented simultaneously but in a given block of trials pressing only one of the two levers would result in a reward. The identity of the rewarded lever reversed after 10 to 32 rewarded trials. After 3 successive sessions of receiving over 100 total rewards and choosing the rewarded lever over 60% of the trials, the mice progressed to the full probabilistic reversal learning task. In the full version of the task, in a given block of trials, one lever was associated with 70% reward probability following a press (high-probability lever) while the opposite lever was rewarded with 10% probability (low-probability lever). The identity of the rewarded lever reversed after 10 to 32 high-probability lever choices. The final choice resulted in an immediate change in contingency. As the outcome of that trial is in the next block, this trial is by definition classed as incorrect. Animals were trained on the full version of the task until they reached the 'expert' level with the consistent performance of over 60% high-probability lever choices for 3 consecutive sessions. While training for either the deterministic or probabilistic stage of the task, mice were also habituated to having optic fibers attached to the implanted ferrules or carrying a dummy Miniscope attached to the implanted baseplate until they met the performance criteria of the corresponding stage. Miniscope or photometry recording experiments commenced after mice were fully habituated and met the

performance criteria for the final stage of the task.

Behavioral analysis

To estimate the number of trials taken by mice to switch their choices to a different lever after reward contingencies reversal, we fit the exponential curve to animals' reversal behavior (proportion of high probability lever presses following the reversal). The fit then allowed us to directly estimate the number of trials taken before animals started choosing the new high probability lever 50% of total lever presses after reversal.

To quantify the influence of the past choice and reward history on animal's choice on the current trial, we used a logistic regression model (*27–29*, *53*). In this model, the target variable was represented by the probability of the current choice being the right lever press (P(i), 1 if right choice, 0 if left choice). Predictor variable consisted of 3 types of trial history regressors: R(i – j) is the rewarded choice history on trial i – j (1 if rewarded right choice, –1 if rewarded left choice, 0 otherwise), N(i – j) is the unrewarded choice history (1 if unrewarded right choice, –1 if unrewarded left choice, 0 otherwise), C(i – j) is the outcome-independent choice history on trial i – j (1 if right choice, –1 if left choice, 0 otherwise). The encoding model is:

$$log\frac{P(i)}{1-P(i)} = \sum_{j=1}^{n}\beta_j^R R(i-j) + \sum_{j=1}^{n}\beta_j^N N(i-j) + \sum_{j=1}^{n}\beta_j^C C(i-j) + \beta_0 \quad (1)$$

where $\beta_j^R, \beta_j^N, \beta_j^C$ are the regression weights of each history predictor, and β_0 is the history-independent constant bias term. While this regressor set is not strictly orthogonal, due to the inclusion of a separate choice predictor, it provides a biologically informed estimate of the contribution of choice and outcome on upcoming choice (*29*).

To model the animal's choice given its trial history, the regression coefficients were fit using LogisticRegression function of *scikit-learn* Python library. For this model we used elastic net regularization, method that combines L1 and L2 regularization penalties to minimize the objective function. First, we performed grid search over C (inverse of the regularization strength) and λ (L1-ratio) hyperparameter space to find the optimal combination of C and λ that explained the most variance when verified with 5-fold cross-validation. The overall total explained variance of the final model R^2 was calculated as an average from 5 cross-validated fits of the model with the best estimated C (0.25 ± 0.04) and λ (0.34 ± 0.04) hyperparameters. C and λ that provided the best average R^2 score were then used to refit the full data set to obtain estimated regression weights. The logistic regression coefficients were fit separately for each session in each animal. Estimated coefficients represented the extent the different past trial choices and outcomes predicted animals' current choices. Model β coefficients were then used to estimate how much different past trial history predictors influenced animals' decisions on the current trial.

Models

We investigated behavioral strategies mice might use when solving the probabilistic reversal learning task by fitting a range of different computational models to their choices. We considered a number of 'simple' models such as random choice, win-stay-lose-shift (WSLS) and choice repetition (*54*) as well as more complex value updating and state inference strategies.

Simple behavioral models:

Random choice: Random choice model assumes that mice do not engage with the task and press levers at random with a bias (b) for one option over the other. The probabilities of choices a and a' on trial t is:

$$p_t(a) = b \tag{2}$$

$$p_t(a') = 1 - b \tag{3}$$

Win-stay / Lose-switch: Noisy WSLS model repeats rewarded actions and switches away from unrewarded actions with probability $1 - \varepsilon/2$ and chooses the other option (switching after rewards, staying after losses) with probability $\varepsilon/2$ The probability of choosing option a is:

$$p_{t+1}(a) = \begin{cases} 1 - \varepsilon/2, & \text{if } (c_t = a \text{ and } r_t = 1)\ OR\ (c_t \neq a \text{ and } r_t = 0) \\ \varepsilon/2, & \text{if } (c_t \neq a \text{ and } r_t = 1)\ OR\ (c_t = a \text{ and } r_t = 0) \end{cases} \tag{4}$$

where c_t is the choice at trial t, and r_t the reward at trial t.

Choice kernel: The choice kernel model tries to capture the tendency for mice to repeat their previous actions. Specifically, the agent computes a 'choice kernel', $CK_t(a)$, for each action, which keeps track of how frequently that option was chosen in the past.

The choice kernel updates according to the rule below:

$$CK_{t+1}(a) = CK_t(a) + \alpha_c(c_t^a - CK_t(a)) \tag{5}$$

where $c_t^a = 1$ if lever a is chosen on trial t, otherwise $c_t^a = 0$, and α_c is the choice kernel learning rate. In the choice kernel model each option is chosen according to a softmax function:

$$p_{t+1}(a) = \frac{\exp(\beta_c \times CK_t(a))}{\exp(\beta_c \times CK_t(a)) + \exp(\beta_c \times CK_t(a'))} \tag{6}$$

where β_c is the inverse temperature associated with the choice kernel.

Value updating (Q) models:

Q-learning: Value updating models are reinforcement learning (RL) models that utilize Q-learning updating rule. In such models on every trial t the expected value $Q_t(a)$ of a chosen action a is updated by the reward prediction error (RPE), the difference between the choice outcome r_t and previous expected value, scaled by the learning rate α:

$$Q_{t+1}(a) = Q_t(a) + \alpha(r_t - Q_t(a)) \tag{7}$$

The choice probabilities were estimated based on the action values according to a softmax function:

$$p_{t+1}(a) = \frac{\exp(\beta \times Q_t(a))}{\exp(\beta \times Q_t(a)) + \exp(\beta \times Q_t(a'))} \tag{8}$$

where β is the inverse temperature.

Supplemented Q-learning models:
Other models from the Q-learning family were augmented alterations of the basic model above.

Q-learning with bias: Introducing bias captures an animals' preference towards one of the levers in the task.

Bias parameter b $(-1 < b < 1)$ changes the expected value of one of the actions reducing or increasing the probability of choosing that action:

$$p_{t+1}(a) = \frac{\exp(\beta \times (Q_t(a) + b))}{\exp(\beta \times (Q_t(a) + b)) + \exp(\beta \times Q_t(a'))} \tag{9}$$

Q-learning with choice kernel: Q-learning strategies may also be affected by animals' tendency to repeat previously selected actions. To incorporate this into the model, we added the choice

kernel (eq. 5 and 6) into the softmax decision rule:

$$p_{t+1}(a) = \frac{\exp(\beta \times Q_t(a) + \beta_c \times CK_t(a))}{\exp(\beta \times Q_t(a) + \beta_c \times CK_t(a)) + \exp(\beta \times Q_t(a') + \beta_c \times CK_t(a'))} \quad (10)$$

Q-learning with asymmetric updates: Reward and punishment (R/P model) sensitivity augmentation utilizes the same value updating rule while using different learning rates following rewarded (α_r) and unrewarded (α_{ur}) outcomes (*29*, *55*):

$$\alpha = \begin{cases} \alpha_r, & \text{if } r_t = 1 \\ \alpha_{ur}, & \text{if } r_t = 0 \end{cases} \quad (11)$$

Q-learning with counterfactual updating: In *counterfactual updating models*, a reward resulting from one choice both increases the expected value of that choice, but also decreases the expected value of the alternative choice (and vice versa following reward omission). In other words, the two choices are interdependent. As a result, such models are often described as an approximation of hidden state inference (*56*), as on each trial the agent infers a change in the value of the unexperienced option (see next section for discussion). In these models, expected values for both actions are updated on every trial: values of the unchosen actions a' are updated according to the counterfactual outcome (1 - r_t) from the chosen action (a) (*5*, *12*):

$$Q_{t+1}(a') = Q_t(a') + \alpha((1 - r_t) - Q_t(a')) \quad (12)$$

We tested four versions of the counterfactual updating models that utilized different sets of learning rates for updating the action values of the chosen and unchosen options following different trial outcomes:

Model	Chosen action		Unchosen action	
	Rewarded trial	Non-rewarded trial	Rewarded trial	Non-rewarded trial
Same a; R/P = False	a	a	a	a
Same a; R/P = True	α_r	α_{ur}	α_r	α_{ur}
Different a; R/P = False	a	a	a'	a'
Different a; R/P = True	α_r	α_{ur}	α'_r	α'_{ur}

Q-learning with forgetting: In value updating models with *forgetting*, expected value of the nonchosen action a' was either directly reset over one trial to the average value $\overline{Q_t}$ across both actions (Forget reset) or gradually updated towards the average $\overline{Q_t}$ according to the forgetting factor δ (Forget gradual):

$$Q_{t+1}(a') = \begin{cases} (1-\delta) \times Q_t(a'), & \text{if } Q_t(a') > \overline{Q_t} \text{ and } (1-\delta) \times Q_t(a') \geq \overline{Q_t} \\ (1+\delta) \times Q_t(a'), & \text{if } Q_t(a') < \overline{Q_t} \text{ and } (1+\delta) \times Q_t(a') \leq \overline{Q_t} \\ \overline{Q_t}, & \text{if } Q_t(a') > \overline{Q_t} \text{ and } (1-\delta) \times Q_t(a') \leq \overline{Q_t} \\ \overline{Q_t}, & \text{if } Q_t(a') < \overline{Q_t} \text{ and } (1+\delta) \times Q_t(a') \geq \overline{Q_t} \end{cases} \quad (13)$$

Q-learning with dynamic value updating: *Dynamic value updating models* are based on the basic RL strategy that utilizes the Pearce-Hall rule (*57*, *58*). These models contain an associability parameter that modulates the learning rate as a function of the absolute magnitude of past RPEs. The κ parameter modulates the action value updating and is equivalent to the learning rate parameter in the basic Q-learning models. On the first trial, α_t is a free parameter. The γ parameter controls the temporal dynamics of associability over time:

$$Q_{t+1}(a) = Q_t(a) + \kappa \times \alpha_t(r_t - Q_t(a)) \quad (14)$$

$$\alpha_t = \alpha_{t-1}(1-\gamma) + \gamma \times |\text{RPE}_{t-1}| \quad (15)$$

where we noted that |RPE| <= 1 and therefore α remains appropriately bounded. Like other value updating models, dynamic value updating models could also be modified to include bias, perseverance, and R/P as described above. R/P is enabled by having different κ parameters for rewarded and unrewarded trial outcomes:

$$\kappa = \begin{cases} \kappa_r, & \text{if } r_t = 1 \\ \kappa_{ur}, & \text{if } r_t = 0 \end{cases} \quad (16)$$

State inference (SI) models:

State inference: State inference models use Bayesian inference and assume that on each trial mice chose their actions based on their belief $b_t(s) = p(s_t|o^{t-1})$ about the underlying state of the task s_t given the history of observations o^{t-1}. In this formulation, action-reward pairs on a given trial are treated as simple observations: $o_t = \{a_t, r_t\}$.

The belief variable takes on a role similar to the Q value in the standard Q-learning models above, becoming a function of the past observations and the parameters (*12*):

$$p(s_{t+1}|o^t) = p(s_{t+1}|s_t) \times \frac{p(o_t|s_t)p(s_t|o^{t-1})}{p(o_t|s_t)p(s_t|o^{t-1}) + p(o_t|s'_t)p(s'_t|o^{t-1})} \tag{17}$$

where $p(o_t|s_t)$, the probability of an observation o_t at trial t, is defined by its 'compatibility' with the state s_t, using parameter c:

$$p(o_t|s_t) = \frac{1}{2} + \frac{1}{2} \times \begin{cases} +c, & \text{if } a_t = s_t \text{ and } r_t = 1 \\ -c, & \text{if } a_t \neq s_t \text{ and } r_t = 1 \\ -c, & \text{if } a_t = s_t \text{ and } r_t = 0 \\ +c, & \text{if } a_t \neq s_t \text{ and } r_t = 0 \end{cases} \tag{18}$$

and the transition probability of the state $p(s_{t+1}|s_t)$ is parameterized by a single parameter γ - the probability of staying in a state:

$$p(s_{t+1}|s_t) = \begin{bmatrix} 0.5 + 0.5\gamma & 0.5 - 0.5\gamma \\ 0.5 - 0.5\gamma & 0.5 + 0.5\gamma \end{bmatrix} \tag{19}$$

Finally, the current belief $b_t(s)$ about the state of the task based on past observations is mapped into action probabilities via a softmax function as in Q-learning models. Note that steepness of the sigmoid is fixed to 10 as this trades off with the state estimate (*12*).

$$p_{t+1}(a) = \frac{\exp(10 \times b_t(s))}{\exp(10 \times b_t(s)) + \exp(10 \times b_t(s'))} \tag{20}$$

Supplemented state inference models: Similar to Q-learning agents, SI models were also supplemented with choice kernel and bias terms. In addition, similar to the R/P augment in the Q-learning models, in the state inference model with reward and punishment sensitivity, different probability parameter d may be used following reward omission:

$$p(o_t|s_t) = \frac{1}{2} + \frac{1}{2} \times \begin{cases} +c, & \text{if } a_t = s_t \text{ and } r_t = 1 \\ -c, & \text{if } a_t \neq s_t \text{ and } r_t = 1 \\ -d, & \text{if } a_t = s_t \text{ and } r_t = 0 \\ +d, & \text{if } a_t \neq s_t \text{ and } r_t = 0 \end{cases} \tag{21}$$

In most fits to animals' behavior the reward omission update parameter d was estimated to be very close to 0, therefore we also tested a model fixing parameter d at 0. Thus, following reward omission $p(o_t|s_t)$ is 0.5 for both actions.

Model fitting

To estimate the values of the parameters that best describe the behavioral data, we used likelihood maximization approach to model fitting (*54*). For this, for each behavioral session we estimated the probability of individual choices based on a given model (m), parameters of the model (Θ_m) and choice and outcome history in that session. We then summed the logs of choice probabilities that corresponded to animals' choices on every given trial. The python function *scipy.optimize.minimize* was used to find the set of parameter values that minimized the negative log of the likelihood of the data (LL) given the model parameters $p(d_{1:T}|\Theta_m, m)$:

$$LL = \log p(d_{1:T}|\Theta_m, m) = \sum_{t=1}^{T} \log p(c_t|d_{1:t-1}, \Theta_m, m) \tag{22}$$

To avoid finding the local minima in the minimization procedure, we repeated model fitting procedure 50 times using randomly selected initial values from defined bounds for each parameter, and recorded the best fitting log likelihood for each run. The best fitting parameters were selected from the run with the highest log-likelihood value.

To determine which model provided the most parsimonious fit to the data we compared different model fits to each individual session using Bayesian Information Criterion (BIC). BIC has an explicit penalty for the number of free parameters (k_m) in the model m and thus controls for overfitting:

$$BIC = -2log\widehat{LL} + k_m log\,(N) \tag{23}$$

where $\widehat{LL}$ is the log-likelihood value at the best fitting parameters, and N is the number of trials in a session. To compare the model fits for each animal, we then computed the differences between the BIC scores of each model fit to individual behavioral sessions with the BIC score of the most parsimonious model of the same session (ΔBIC). In fig. S3 we additionally calculate an alternate ΔBIC where all model fits to each session are compared explicitly to the SI model used in the main figures.

Determination of example models for main comparison

According to the BIC analysis, all behavioral sessions from all mice were best described by SI models. Specifically, the most parsimonious SI models had reward and punishment sensitivity (R/P) and used only rewarded trials for $p(o_t|s_t)$ updates ($d = 0$); while some also had choice bias. This means that the 'best' SI model uses $p(o_t|s_t)$, $p(s_{t+1}|s_t)$ and $p(s_t|o^{t-1})$ as part of its estimation of the current state on rewarded trials, but on unrewarded trials $p(o_t|s_t) = 0.5$ and therefore only $p(s_{t+1}|s_t)$ and $p(s_t|o^{t-1})$ influence the belief update. This strategy appears to be common during performance of probabilistic behavior in mice (*60*), and allows a tolerance for high reward omission rates on the correct option, enabling stable performance at relatively low reward probabilities. Among the Q model group, the most parsimonious models either had only supplemented choice bias parameter, or had asymmetric learning rates for rewarded and unrewarded outcomes (R/P) as well as choice bias.

Therefore, for our comparison of the SI and Q models in the main figures, we focused on the versions of Q and SI models that included the same augmentations – R/P and choice bias.

Importantly, we focused on Q-learning without counterfactual updating in the main figures, as – because this model is commonly seen as an approximation of SI (*56*) – we wanted to avoid confounds due to the presence of inference in our main comparisons. However, for completeness we also include comparisons of both behavior (fig. S2) and dopamine (fig. S5) predictions from supplemented Q-learning models (including counterfactual updating, dynamic learning rates and forgetting). In these comparisons we also compared exemplar models with the same augmentations for consistency (R/P and choice bias). These specific models are highlighted in fig. S2 and S3, and details of parameters for each model are outlined in Table S3. In all cases, predictions from SI models were most consistent with the data. The ability of each model to describe data in each part of the study are summarized in fig. S9. To assess changes in behavior in caspase lesion/sham animals (Fig. 3C-E), mouse behavior was fit as above from sessions obtained at baseline before lesion. The effects of the lesion or sham were then assessed using these model parameters to investigate how model predictions of trial-by-trial behavior were altered by the manipulation.

Model simulations

To simulate the probabilistic reversal learning behavior, we ran the models with the parameter sets obtained from the model fits to individual mouse sessions. Each set of parameters was used for 3 simulation runs, and a simulation run comprised of 300 trials. To obtain trial-by-trial choice probabilities from different strategies (Fig. 1H and fig. S1, S2) we used sets of average parameters from model fits to update model predictions based on animals' choices and outcomes on a trial-by-trial basis (*9,30*).

Model verification

To check how reliably we can conclude that the best model from the fitting procedure was more likely to have generated the data compared to other models that were tested we performed model

recovery (*54*). We approached this in 3 stages, directly comparing the Q and SI models used in the main figures (fig. S1), comparing the exemplar models of each class (fig. S2), and finally comparing all models with all supplements (fig. S3). For each of these analyses, we used simulated data from all models and fit that data with all models. From this we quantified the proportion of the simulated data generated by one model that was best fit other models p(fit model|simulated model), summarized in a confusion matrix. If 100 % of the simulations were best fit by the same models that produced the simulated data, the confusion matrix would be the identity matrix. We also computed the inversion matrix that quantified the probability the model generated the data given that it provided the best fit p(simulated model|fit model). From the inversion matrix we can estimate the confidence with which we can draw conclusions about the behavioral strategies based on the best fitting models (how likely the same model is to have generated the data). As evidenced from these figures (and in particular fig. S2 and S3), while there was confusion within class, for example between different implementations of Q-learning; there was very little confusion across strategies (see fig. S1). Therefore the model fitting approach allows investigation of Q vs SI strategies in mouse behavior.

Photometry

Recording setup. To measure dopamine release, we recorded dLight1.1 fluorescence using a custom-built fiber photometry acquisition as described previously (*46*, *51*). Briefly, to record dLight-dependent fluorescence we used blue 470 nm LED, while to control for dopamine-independent fluctuations in recorded fluorescence (e.g. due to movement) we used violet 405 nm LED. LEDs were controlled via a custom script written in LabView (National Instruments). To enable synchronization with the behavioral task, the recording was initiated by a TTL pulse from the MED-PC program at the start of the behavioral session. To ensure the separation of the blue and the violet channels, the light amplitudes were modulated sinusoidally with two different frequencies (500 Hz and 210 Hz, respectively). For excitation, light from both LEDs passed through corresponding excitation filters (470 nm and 405 nm) before being combined into a single beam by a dichroic mirror. The excitation light was then passed through a beam splitter to allow for simultaneous recordings in two animals. The excitation beams were then reflected off a dichroic mirror, collimated and launched into a fiber patch cord (200 µm core and 0.22 NA). The patch cord was connected to the ferrule of the implanted optical cannula on the animal's head via an interconnect. The emission signal was passed through the same patch cord and collimator, and filtered through an emission filter (transmission above 505 nm). It then passed through a dichroic mirror and focused onto a femtowatt photoreceiver (Newport) sampling at 10 kHz. Each of the two modulated signals generated by the two LEDs was recovered using standard demodulation techniques implemented by a custom Labview script. dLight and control autofluorescence signals were then downsampled to 500 Hz before being exported for further analysis.

Photometry data processing. Photometry data were analyzed with custom-written Python scripts (*46*, *51*). First, to reduce the noise, a lowpass filter was used on both dLight and control signal. To correct for photobleaching, a 4th order polynomial fit was subtracted from each trace. The fluorescent signal obtained after stimulation with control 405 nm LED was used to correct for dopamine-independent changes in fluorescence such as due to movement. Movement artifacts were estimated by a least-squares linear fit of the control signal from 405 LED excitation to the

dLight fluorescence. The estimated movement signal was then subtracted from the dLight signal to obtain the movement-corrected signal corresponding to dopamine release. Signals were then z-score normalized. For photometry experiments with chronic taCasp3 hippocampal inactivations, we excluded data from mice where dLight signals were not observable (2 mice from mCherry control group), or where mice had misplacement of caspase injections inferred from the immunohistochemistry labelling (2 mice from taCasp3 group).

Photometry data analysis. Dopamine signals were analyzed in two complementary ways. First, z-scored signals were aligned to C+ or C- and baselined to the mean signal from 1 s preceding the event. The event summary was obtained by calculating mean of the baselined signal in the first 4 seconds of the event. Selection of a wide time window for the event summary enabled us to capture most of the event-associated signal in an unbiased way irrespective of the temporal variability across animals. These events were then sorted according to past choice and outcome history (same or opposite choice, rewarded or non rewarded outcome), and compared to RPE calculated from simulations of agents utilizing Q or SI strategies. For Q estimates, RPE on a particular trial was the outcome minus the estimated value for that choice. For SI estimates, RPE was the outcome minus the estimated outcome compatibility given state (Eq. 21). We compared model estimates qualitatively across pairs of choices and outcomes (fig. S2), but also investigated this more quantitively using a regression approach to predict dLight signal across each of the 8 trial types using either SI and Q RPE as predictors. Second, we used 2-fold cross validated ridge regression to express dLight fluorescence as a sum of responses related to outcome, past outcome, choice and past choice, as well as estimates of Q-RPE and SI-RPE from our model fits. For this analysis, photometry traces were aligned across trials by linearly time-warping the signal at the intervals between different fixed trial events and resampling the signal at a fixed rate. We only included data within 6 trials of a switch in contingency, due to the increased number of incongruent trials allow better discrimination of the two RPE predictors. Behavioral predictors (outcome, past outcome, choice and past choice) were binary variables centered at 0 (i.e. outcomes were coded as 0.5 for rewarded and -0.5 for non-rewarded), while latent variables (SI- and Q-RPE) were continuous estimates from model fits. For each time point we calculated the coefficient of partial determination (CPD) for each predictor, i.e., what percentage of the variance of the dLight activity at that time-point was explained by the full regression analysis that was not explained by the regression analysis if that predictor was removed. To complement this we also performed single predictor regressions where we calculated the variance that could be explained by only one predictor. For both of these metrics we compared the contribution of SI- and Q-RPE to model fits, and the influence of vCA1 lesions.

Miniscope

Recording setup. Calcium imaging was acquired using Miniscope V4 – a head-mounted microscope (OpenEphys) controlled via Miniscope-DAQ-QT-Software. A blue LED was used for excitation (~470 nm spectral peak) with power adjusted to approximately match the mean brightness of the image across animals. Fluorescence was passed through an emission filter (bandpass filter, 525/50 nm) and collected by a CMOS imaging sensor. Before the start of the recording, the Miniscope was attached to the base plate and its focal plane was adjusted. Afterwards, the mouse with the Miniscope attached was placed in a behavioral chamber (MED

Associates, ENV-307W) for 3–5 minutes before the recording session started. Miniscope was connected to a laptop via a flexible coaxial cable and an off-board data acquisition (DAQ) board and the calcium imaging data was acquired at 30 Hz using Miniscope-DAQ-QT-Software (https://github.com/Aharoni-Lab/Miniscope-DAQ-QT-Software#miniscope-daq-qt-software).

Miniscope data processing. Minian software was used for all pre-processing stages and subsequent fluorescence signal extraction (*61*). To improve the computational performance of the processing pipeline, the videos were first cropped to a rectangle containing the imaged cells, the video width and height was down sampled by a factor of 2, and the framerate was down sampled by a factor of 2. Following the correction of the background fluorescence and median filtering for sensor noise removal, the video was motion-corrected and seeds for estimation of cells' spatial footprints were initialized. This set of seeds was then used for cell and signal detection using a constrained non-negative matrix factorization (CNMF) algorithm (*62*). Following the refinement of the spatial footprints and denoising of the temporal traces of each cell, the CNMF algorithm produced background-subtracted calcium fluorescence values and deconvolved the calcium trace into estimated 'spikes' that corresponded to a scaled probability of neural activity. The results were then visually inspected and non-cell like shapes and traces were excluded from the output. Deconvolved calcium traces were subsequently aligned across trials by time-warping as described for photometry above.

Selectivity index analysis. Trial type selectivity of individual neurons was computed as:

$$SI = \frac{f_+ - f_-}{f_+ + f_-} \tag{24}$$

where f_+ and f_- are the average activity of the neuron in the period from 1s before trial initiation up to the C+ or C- delivery on different trial types (right vs left, high or low reward probability, state A vs state B choices) (*63*). To assess the statistical significance of selectivity indices (SIs) of individual neurons, we compared their SI values to those derived from 1000 shuffled datasets, where the labels of trial types were randomly reassigned.

Neuronal trajectory analysis. The neural population activity trajectories were obtained by projecting the average population activity for each trial type into the low dimensional space that captured most variance between trial types. Every trial in the task belonged to one of four conditions defined by the combination of animal's choice and reward contingency associated with the chosen lever in a current block of trials: left-high (LH), right-high (RH), left-low (LL), and right-low (RL). First, to evaluate the component of activity that was not selective to different trial types, we calculated the average activity for each neuron across all trial types. We then subtracted the non-selective activity for each neuron from that neuron's average activity for each individual trial type, baselined to isolate within-trial variation, and concatenated across trial types to generate a data matrix representing how activity for each neuron deviated from its cross-trial-type average in each trial type (*64*). We performed PCA on this matrix to find the space that captured the most cross-trial-type variance and then projected the average population activity trajectory for each trial type into this space.

Population decoding analysis. The decoding analysis was used to predict different trial types based on mean spiking probability of simultaneously recorded neurons. For this, average neural responses associated with different task variables we estimated as a mean spiking probability of individual neurons from 1 s before the trial initiation up until C+ or C-. Based on different combinations of animal's choices and associated reward probabilities outlined above, each trial could be classified based on animal's choice (lever identity – LH and LL vs RH and RL), expected outcome (reward probability associated with the chosen lever – LH and RH vs LL and RL) or task state (a set of reward contingencies associated with either choice in a given block of trials – LH and RL vs LL and RH). To balance the number of different trial types, for each neuron each class trial pool was randomly sampled 250 times with replacement. Unless states otherwise, the decoding analysis was performed on activity from simultaneously recorded neurons from a single behavioral session.

For decoding, we used a support vector machines (SVM) classifier with a linear kernel implemented through LinearSVC function from *scikit-learn* Python library. For cross-validation, data were randomly divided into two non-overlapping groups of trials, used for training and testing the classifiers (75/25% split). The decoder was trained to discriminate between population responses corresponding to two sets of trial variables representing either animal's choice, expected outcome, or task state. The regularization hyperparameter C was optimized using GridSearchCV *scikit-learn* function with 5-fold cross-validation. The decoding performance was then tested using a held-out test set. This procedure was repeated at least 100 times for each classifier with random train/test subdivisions and the decoding accuracy was computed as the average result across repetitions. To assess the statistical significance of the decoding accuracy, we repeated the same procedure described above on a dataset with shuffled trial labels.

For cumulative decoding plots, we generated neural pseudo-populations from subsets of neurons sampled across multiple animals and/or recording FOVs. Each trial condition was sampled 250 times and activity of individual cells within that condition shuffled. The decoding analysis was performed as for single session models outlined above.

Generalization decoding analysis. To quantify the degree of generalized representation of the trial variables, we used cross-condition generalization performance (*65*). In generalization decoding analysis training and testing sets were created by splitting trials according to their trial labels, so the decoder was trained to discriminate trial categories according to half of the labels and then the discrimination generalization was assessed on the data from different conditions not used in training. For example, to test generalized encoding of choice, the decoder was trained to discriminate RH and LH trials and its performance was tested on discrimination of RL and LL trials, respectively.

Statistical analysis

All statistics were calculated using the Python packages *scipy, pingouin* and *statsmodels*, and *lme4* R package implemented in Python through rpy2. Summary data are reported as mean ± SEM (standard error of the mean). Unless otherwise stated, statistical tests were performed comparing data from individual behavioral sessions, including mouse identity as a random effect to maintain

the dependence between sessions from individual mice. As a result, p values are estimated using the Satterthwaite approximation. Normality of data distributions was determined by visual inspection of the data points. Test statistics are detailed in the supplementary statistics table. Threshold for statistical significance was defined as 0.05. Animals were randomly assigned to a virus cohort (e.g. sham versus lesion), and as far as possible, littermates testing each variable of interest were present in each cohort to control for experiment-to-experiment variability. The experimenter was not blinded to each mouse's assignment. No power analysis was run to determine sample size a priori. The sample sizes chosen are similar to those used in previous publications. Throughout the figures the * symbol represents $p < 0.05$.

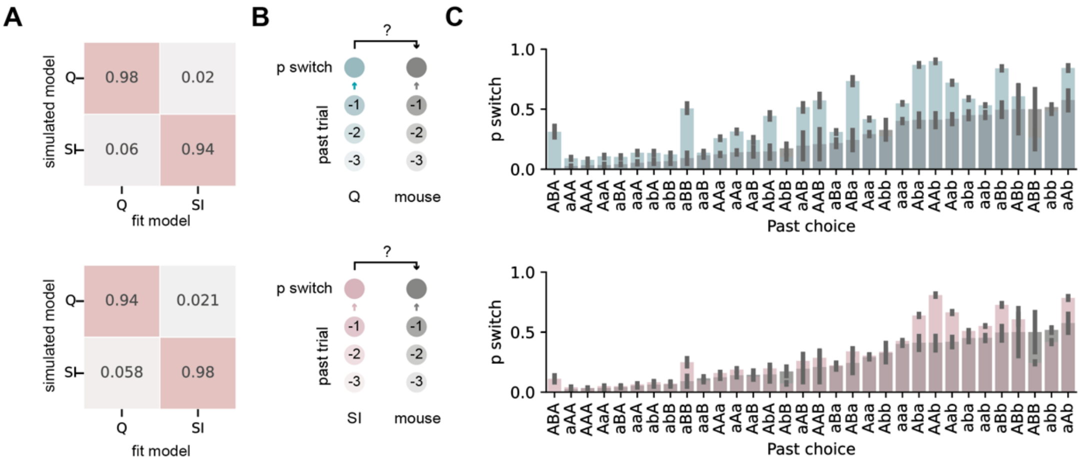

Fig. S1. Further characterization of Q and SI model fits of mouse behavior.

(**A**) Confusion (top) and inversion (bottom) matrix showing Q and SI models are distinct and readily distinguishable. (**B**) Schematic of comparison of model predictions with mouse choices after distinct 3-trial histories. (**C**) Mouse switching behavior (grey) split by past choice and outcome history. Different combinations of the past three choices (A or B) and whether they were rewarded (A, B) or not rewarded (a, b) are plotted on the x axis, and probability of switching choice on the next trial is plotted on the y axis (as shown in (*9,30*)). Q (top, blue) and SI (bottom, pink) model switch probability estimates for the same trial histories are overlayed, note SI prediction is most similar to mouse behavior, quantified in Fig. 1H.

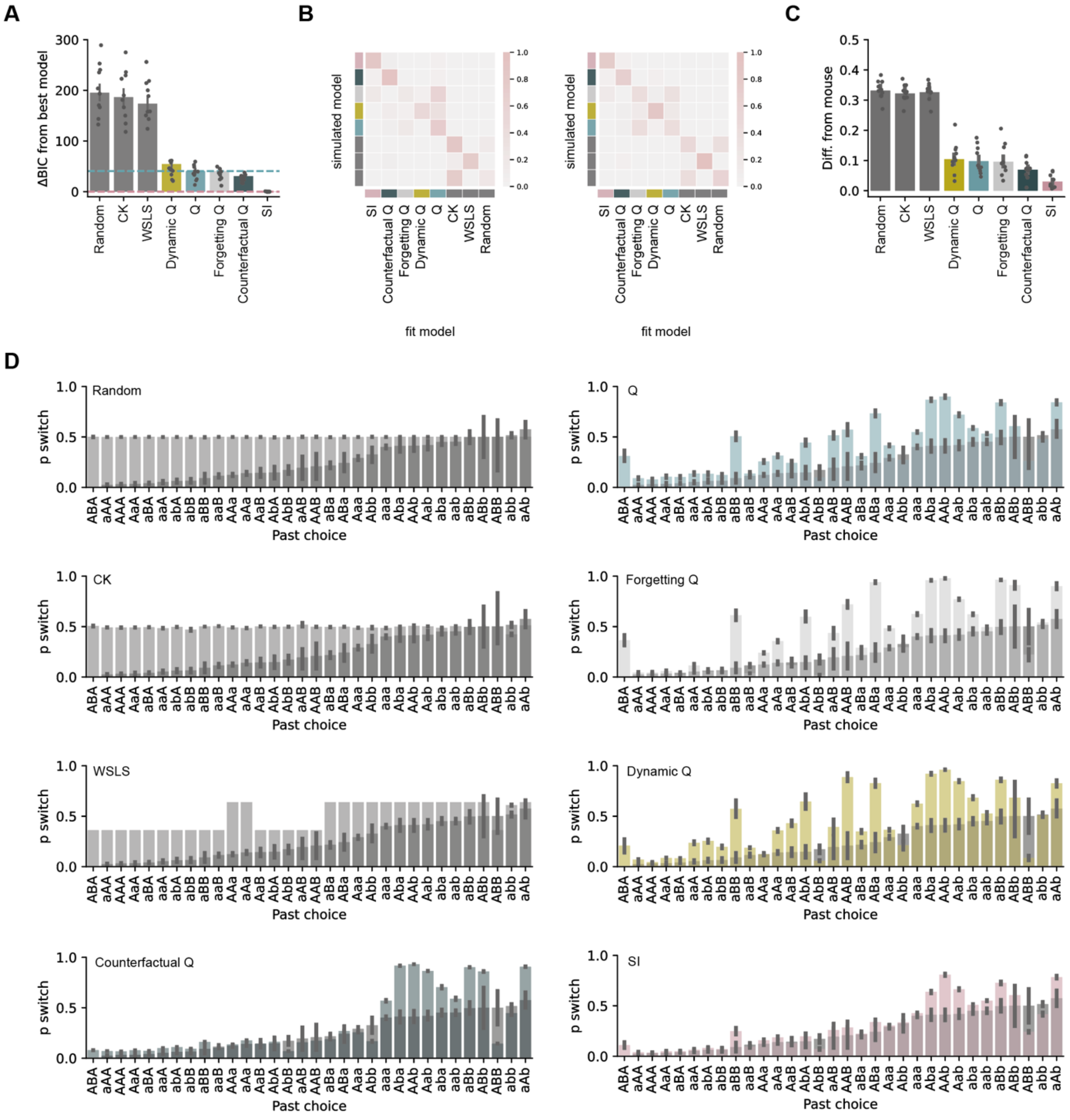

Fig. S2. Characterization of fits from exemplar alternate and supplemented Q-learning models.

(**A**) ΔBIC of model fits compared to the most parsimonious model for each session, averaged across mice. Model types are color coded and include random choice (Random), choice kernel (CK), win-stay/lose-switch (WSLS), Q-learning with dynamic updating (Dynamic Q), unsupplemented Q-learning (Q), Q-learning with forgetting of the unchosen option (Forgetting Q), Q-learning with counterfactual updating (Counterfactual Q), and State inference (SI). See methods for strategy underlying choice of exemplar models. (**B**) Confusion (left) and inversion (right)

matrix for models in (**A**). Note that while there is confusion among models utilizing the same overall strategy (e.g. Q-learning with different supplements can be confused with each other), models using different strategies are distinct and readily distinguishable. (**C**) Summary of difference between mouse switching behavior at different trial histories, and that of simulations utilizing different model strategies, note SI prediction is most similar to mouse behavior. (**D**) Mouse switching behavior (grey) split by past choice and outcome history summarized in (**C**). Different combinations of the past three choices (A or B) and whether they were rewarded (A, B) or not rewarded (a, b) are plotted on the x axis, and probability of switching choice on the next trial is plotted on the y axis (as shown in (*9,30*)). Model predictions of the same trial histories are overlayed.

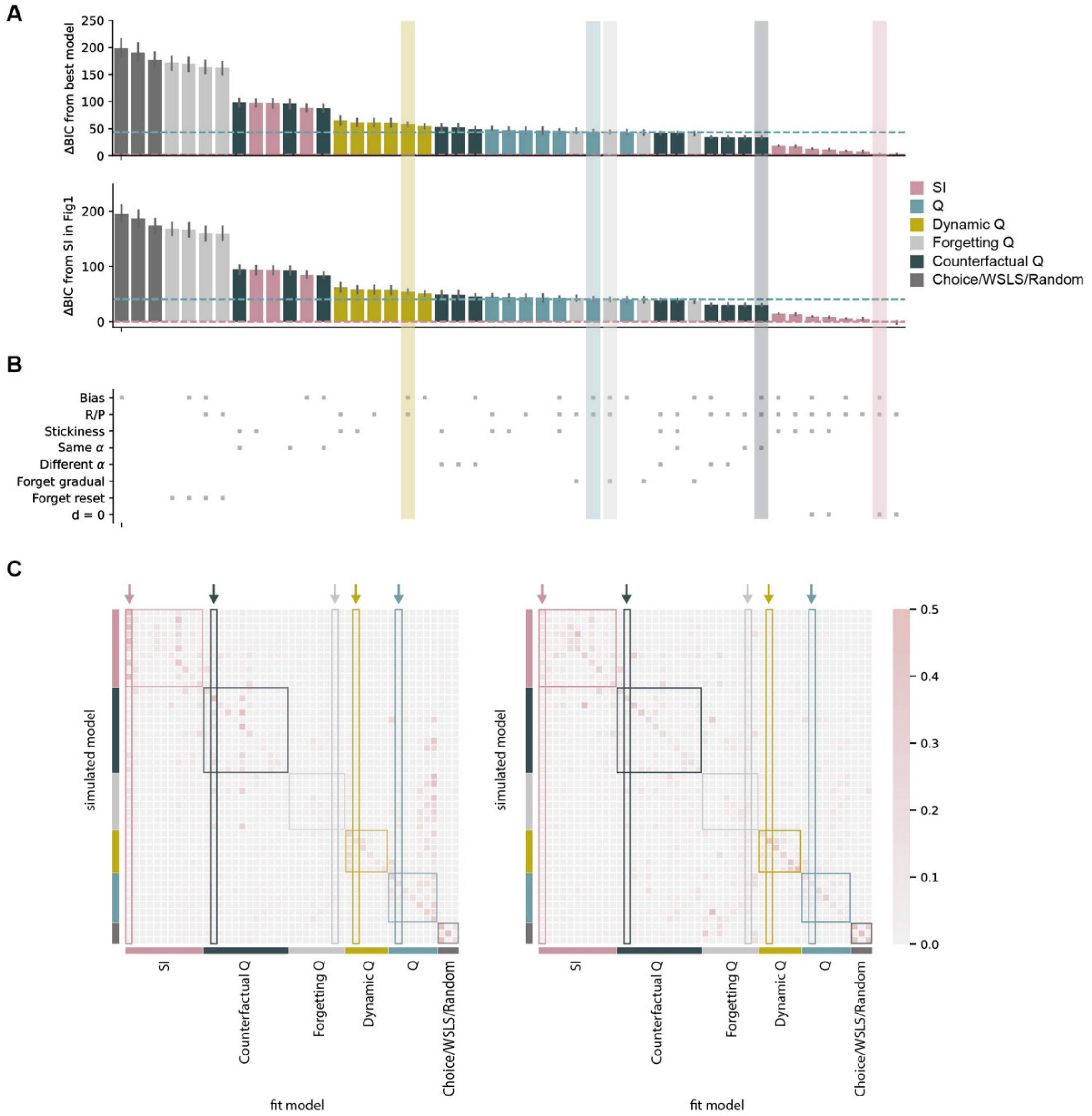

Fig. S3. Characterization of fits from all models.

(**A**) *Top* ΔBIC of model fits compared to most parsimonious model for the same session, averaged across mice. Bottom, ΔBIC of model fits for each session compared to SI model used in main figures. Model types are color coded and include random choice (Random), choice kernel (CK), win-stay/lose-switch (WSLS), Q-learning with dynamic updating (Dynamic Q), unsupplemented Q-learning (Q), Q-learning with forgetting of the unchosen option (Forgetting Q), Q-learning with counterfactual updating (Counterfactual Q), and State inference (SI), each with different versions supplemented with extra parameters outlined in (**B**) and in Table S2. Exemplar models used throughout the manuscript are highlighted. (**C**) Confusion (left) and inversion (right) matrix for models in (**A**). While there is confusion among models – particularly those providing a poor fit to the data, this was most common in models utilizing the same overall strategy (e.g. Q-learning with

different supplements). In contrast, models using different strategies (particularly models that provide a good fit to the data such as the exemplar models highlighted) are distinct and readily distinguishable.

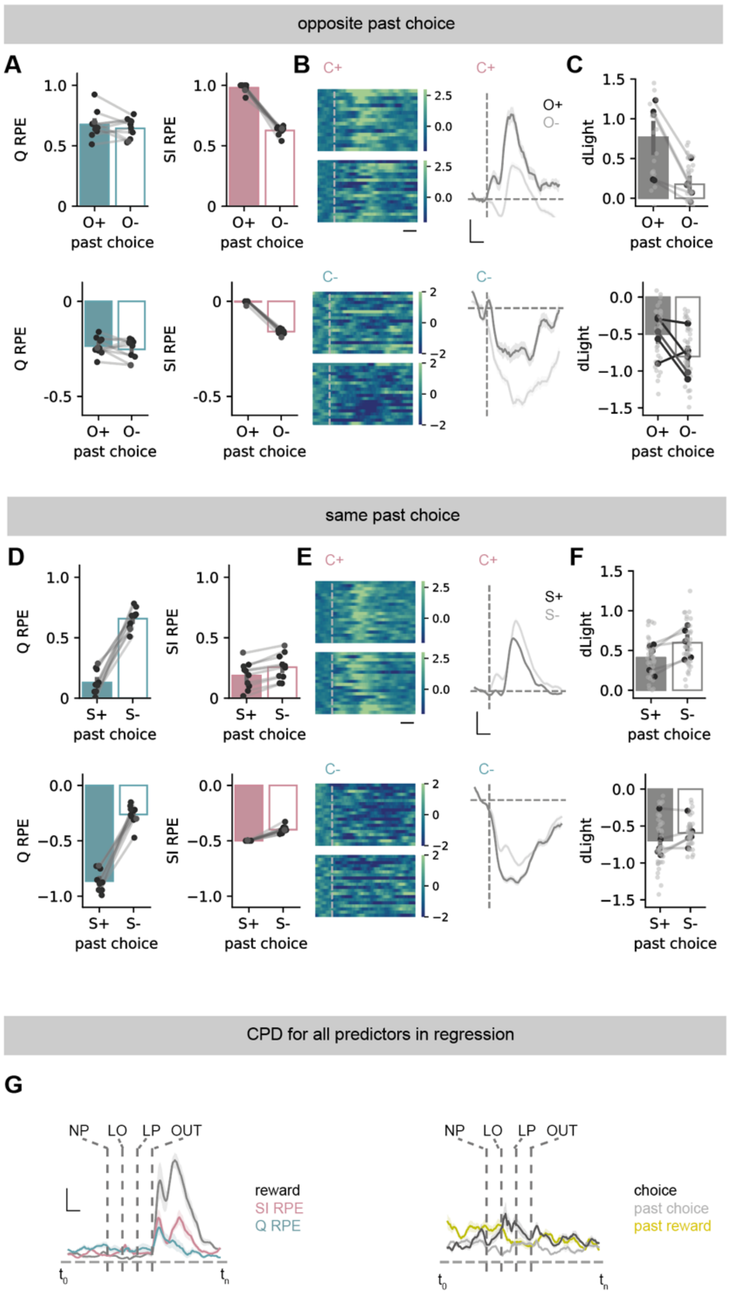

Fig. S4. Dopamine dynamics across different choice and outcome histories.

(**A**) Prediction error estimates from Q (left) or SI (right) models for trials split according by outcome of past opposite lever press (see text): O+ (reward) or O- (reward omission). Top shows current rewarded trials, bottom shows current unrewarded trials. Note only SI predictions change. (**B**) Left, z-scored dLight signal for rewarded trials aligned to cue (C+, top), and unrewarded trials aligned to cue (C-, bottom) for O+ or O- trials. Right, example z-scored dLight signal around C for O+ and O- trials for one session. Scale bar = 1 s (left); 1s, 0.5 zF (right). (**C**) Summary of dLight signal on O+ and O- trials. (**D-F**) As (**A-C**) but split by the outcome of the same past choice.

Note that there is no large prediction error despite occasional non reward trials, consistent with the probabilistic context being known to the animal (**G**) Coefficients of partial determination for top: outcome (grey), Q-RPE (blue), SI-RPE (pink), bottom: choice (black), past choice (grey) and past reward (yellow) at each timepoint. Scale bar = 1 s, 2.5 %. Error bars represent s.e.m. across animals, dark points show individual mice (model simulations n = 10, dopamine recordings n = 5).

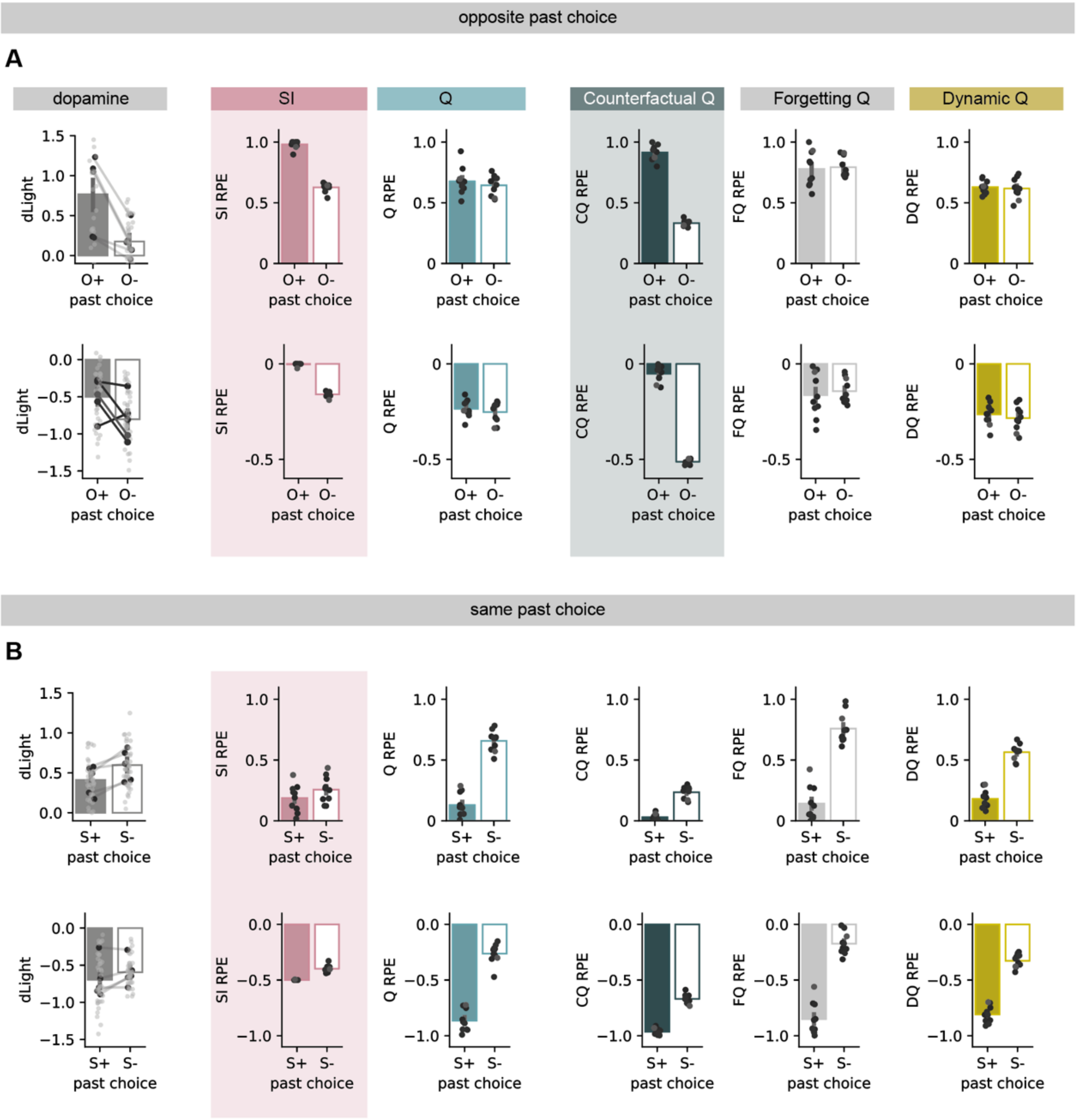

Fig. S5. Predictions of dopamine dynamics from different models across choice and outcome histories.

Prediction error estimates as in fig. S4 from exemplar models outlined in fig. S2. (**A**) *Left*, Summary of dopamine recorded during the task for trials split according by outcome of past opposite lever press (see text): O+ (reward) or O- (reward omission). *Top* shows current rewarded trials, *bottom* shows current unrewarded trials. *Right*, Predictions of exemplar models for the same trial types. Models include state inference (SI, pink) and unsupplemented Q-learning (Q, blue) used in the main text. Additional models are supplemented Q-learning models: Q-learning with counterfactual updating (Counterfactual Q, green), Q-learning with forgetting of the unchosen option (Forgetting Q, grey), and Q-learning with dynamic updating (Dynamic Q, yellow). Model predictions that match dopamine dynamics are highlighted. (**B**) As shown in (**A**) but for past

choices on the same lever (S+ vs S-). Note that SI predictions are a close match to recorded dopamine in all conditions, while Q is not. In addition, while counterfactual Q models predict dopamine dynamics in O+ and O- trials, they do not on S+ and S- trials, and instead follow Q-learning predictions. Other Q-learning supplements do not affect predictions, and remain consistent with predictions from unsupplemented Q. Error bars represent s.e.m. across animals, dark points show individual mice (model simulations n = 10, dopamine recordings n = 5).

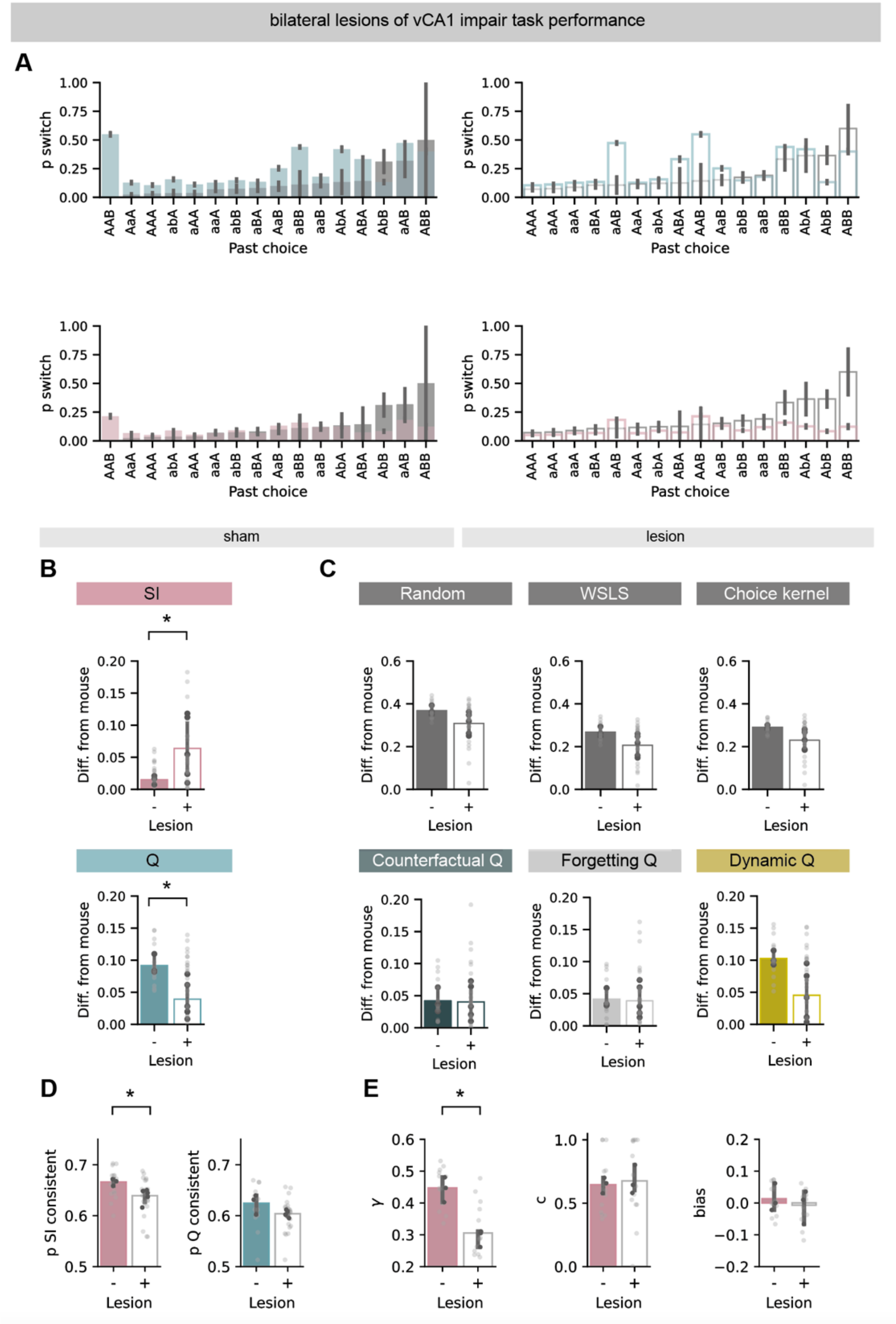

Fig. S6. Further characterization of the influence of bilateral vCA1 lesions on behavior.

(**A**) Mouse switching behavior split by past choice and outcome history with a previous rewarded outcome. Q (top) and SI (bottom) model predictions overlayed on sham (left) and lesioned (right) animals. Summarized in (**Fig. 3C,D**). (**B**) Summary of difference between mouse switching behavior in sham vs lesion animals at different trial histories, and that of simulations utilizing

either SI (top) or Q (bottom) strategies. vCA1 lesions result in a decrease in accuracy of SI predictions, and a coincident increase in accuracy of Q predictions. Same data as in Fig. 3C,D, replotted across groups for clarity. (**C**) As in (**B**) but for predictions from other exemplar models outlined in fig. S2. Note main influence of vCA1 lesion is to alter Q and SI predictions, with minimal effect on other strategies. (**D**) Proportion of choices consistent with SI strategy (left) and Q strategy (right) in sham and lesioned mice around a switch in contingency. Note lesion has limited effect on Q consistent choices. (**E**) SI model parameters for fits to sham and lesions sessions. Error bars represent s.e.m. across animals, dark points show individual mice (model simulations n = 10, sham n = 3, lesion n = 5). *$P < 0.05$.

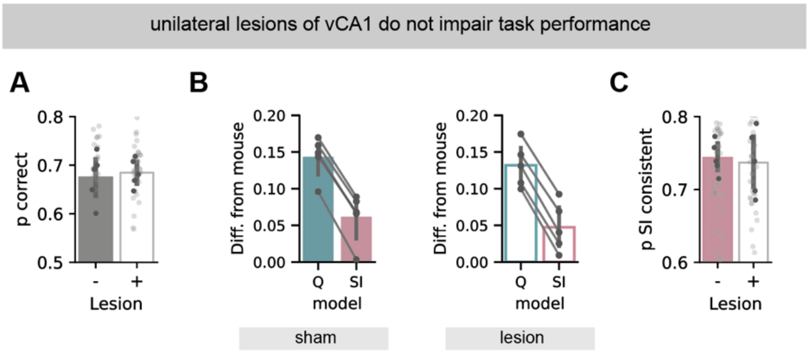

Fig. S7. Characterization of the influence of unilateral vCA1 lesions on behavior.

(**A-C**) Unilateral lesions do not affect behavior: (**A**) Summary of high probability choices in sham and caspase lesioned mice. (**B**) Difference between mouse switching behaviour at different trial histories, and that of simulations utilising either Q or SI strategies in sham and lesioned mice. **(C)** Proportion of choices consistent with SI strategy in sham and lesioned mice. Dark points show individual mice, light points show individual sessions (n = 5 mice per group).

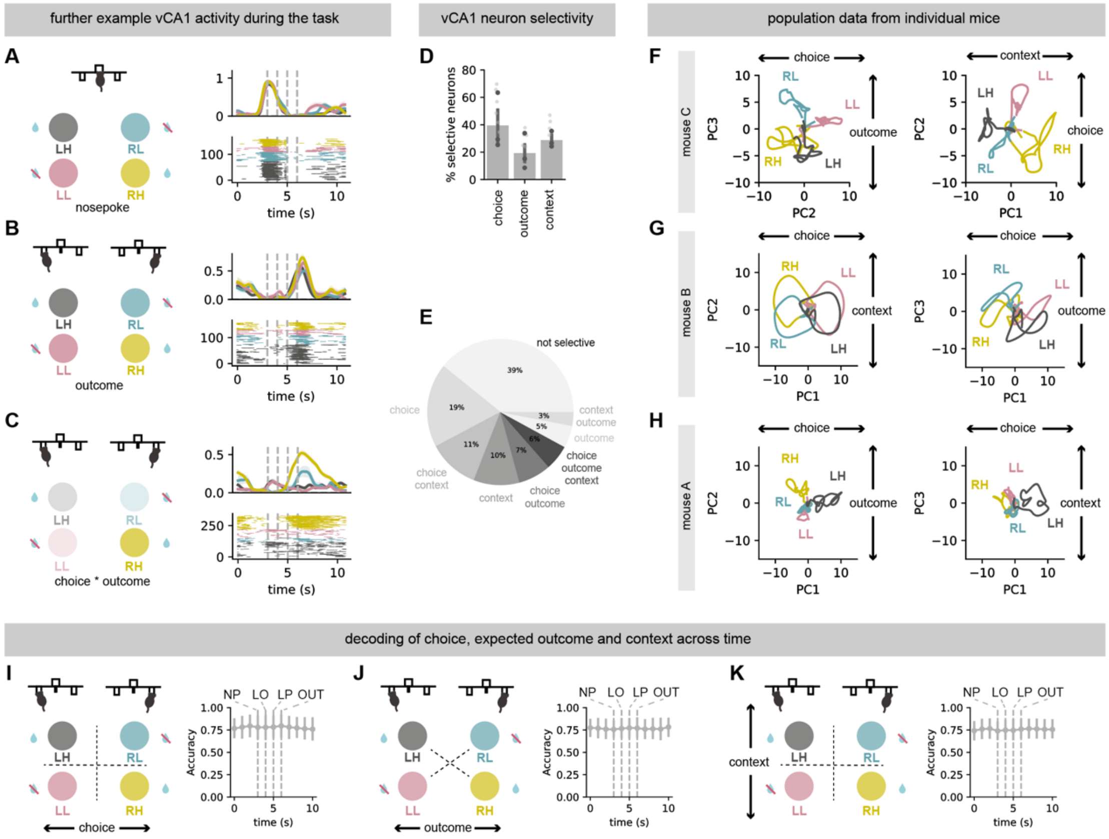

Fig. S8. Analysis of vCA1 activity, trial selectivity and consistency across mice.

(**A**) Example vCA1 neuron with activity preferentially around trial initiation (nosepoke), irrespective of trial type. (**B**) As in (**A**) but neuron tuned to outcome, irrespective of trial type. (**C**) Example neuron with activity preferentially on only RH trials. (**D**) Summary of proportion of neurons selective for either choice, expected outcome or context. (**E**) Proportion of neurons selective for different combinations of choice, expected outcome or context. (**F-H**) Principal components calculated as in **Fig. 4F**, but for individual mice. Note that each mouse has similar separation of choice, expected outcome and context across the first three PCs. (**I-K**) Decoding analysis as in **Fig. 4K**, for choice (**I**), expected outcome (**J**) and context (**K**), but for individual 1s epochs throughout the trial. Note that decoding of each variable is stable across the trial and ITI. Dark points show individual mice, light points show individual sessions.

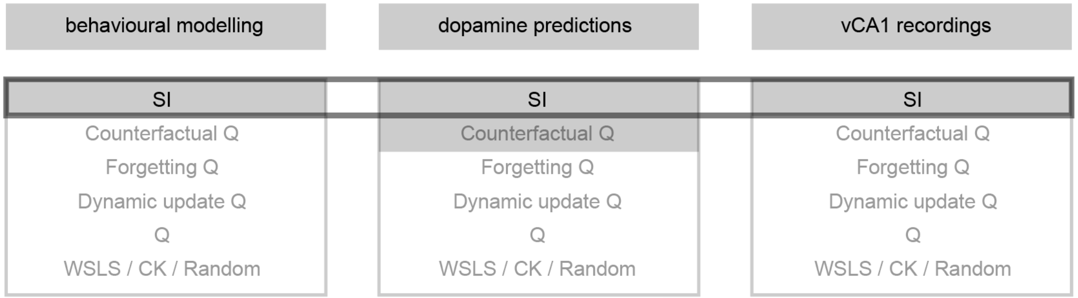

Fig. S9. Summary of how different strategies describe findings.

Summary of key findings. Model fits to behavioral data show that SI provides the best description of mouse behavior. Dopamine recordings show SI models best predict data, and counterfactual Q models provide a description of some key aspects. vCA1 recordings show robust differentiation of latent context suggesting use of SI.

Table S1. Supplementary Statistic Table.

Figure	Descriptors	n	Test used	Statistic	p-value
1B	Reward (R), Choice (C) and Non Reward (N) coefficients (trials -1 to -5)	10 mice	T-test (with Holm Sidak correction)		
	R1			$t_{(9)}$ = 10.37	3.70 x10^{-5}
	R2			$t_{(9)}$ = 9.25	8.88 x10^{-5}
	R3			$t_{(9)}$ = 5.68	0.003
	R4			$t_{(9)}$ = 6.42	0.001
	R5			$t_{(9)}$ = 6.03	0.002
	N1			$t_{(9)}$ = 2.84	0.085
	N2			$t_{(9)}$ = 4.70	0.009
	N3			$t_{(9)}$ = 2.50	0.097
	N4			$t_{(9)}$ = 2.18	0.097
	N5			$t_{(9)}$ = 2.53	0.097
	C1			$t_{(9)}$ = 11.79	1.34 x10^{-5}
	C2			$t_{(9)}$ = 8.97	1.05 x10^{-4}
	C3			$t_{(9)}$ = 4.34	0.013
	C4			$t_{(9)}$ = 3.03	0.085
	C5			$t_{(9)}$ = 2.94	0.085
1G	∆BIC<br>Q vs SI fit	10 mice	Paired t-test	$t_{(9)}$ = 8.76	0.000011
1H	Difference between mouse and prediction from Q vs SI	10 mice	Paired t-test	$t_{(9)}$ = 9.73	0.000005
1I	p choice consistent with<br>Q vs SI	10 mice	Paired t-test	$t_{(9)}$ = 3.55	0.006
2D	Q RPE: O+ vs O-<br><br>SI RPE: O+ vs O-	10 simulations	Paired t-test	$t_{(9)}$ = 1.19<br><br>$t_{(9)}$ = 20.67	0.26<br><br>6.78 x10^{-9}
2F	dLight: O+ vs O-	41 sessions from 5 mice	Paired t-test	$t_{(66.4)}$ = 3.62	0.000574
2G	R^2 of Q vs SI regression	41 sessions from 5 mice	Paired t-test	$t_{(60)}$ = 2.59	0.012

2J	Q RPE CPD vs<br>SI RPE CPD	5 mice	Paired t-test	$t_{(4)}$ = 2.82	0.047
2K	Q RPE alone R^2 vs<br>SI RPE alone R^2	5 mice	Paired t-test	$t_{(4)}$ = 4.34	0.012
3B	p correct:<br>sham vs lesion	Sham: 25 sessions from 3 mice<br>Lesion: 35 sessions from 5 mice	t-test	$t_{(42)}$ = 3.55	0.001
3C,D	Difference between mouse and prediction from Q vs SI<br>Sham vs lesion<br>(log transformed)	Sham: 25 sessions from 3 mice<br>Lesion: 35 sessions from 5 mice	Paired t-test<br><br>Paired t-test<br><br>Mixed effects ANOVA<br>Effect of Model<br>Effect of lesion<br>Interaction	$t_{(30)}$ = 7.21<br><br>$t_{(54)}$ = 0.92<br><br><br>$t_{(84)}$ = 1.08<br>$t_{(84)}$ = 1.68<br>$t_{(84)}$ = 2.18	5.27×10^{-7}<br><br>0.36<br><br><br>0.28<br>0.10<br>0.03
3E	p SI consistent:<br>sham vs lesion	Sham: 25 sessions from 3 mice<br>Lesion: 35 sessions from 5 mice	t-test	$t_{(48)}$ = 3.75	0.0005
3H	dLight: O+ vs O-<br>sham<br>lesion	 41 sessions from 5 mice<br>43 sessions from 5 mice	 Paired t-test<br><br><br>Mixed effects ANOVA<br>Effect of past choice<br>Effect of lesion<br>Interaction	 $t_{(66.4)}$ = 3.62<br>$t_{(71.4)}$ = 0.65<br><br><br>$t_{(137.6)}$ = 4.06<br>$t_{(13.8)}$ = 0.82<br>$t_{(137.8)}$ = 2.53	 0.000574<br>0.52<br><br><br>8×10^{-5}<br>0.44<br>0.01
3I	R^2 of Q vs SI regression<br>sham<br>lesion	 41 sessions from 5 mice<br>43 sessions from 5 mice	 Paired t-test	 $t_{(60)}$ = 2.59<br>$t_{(58.9)}$ = 0.33	 0.01<br>0.74
3J	Q RPE CPD vs<br>SI RPE CPD<br>sham<br>lesion	 <br>5 mice<br>5 mice	 <br>Paired t-test<br><br><br>Mixed effects ANOVA<br>Effect of lesion<br>Effect of p context<br>Interaction	 <br>$t_{(4)}$ = 2.82<br>$t_{(4)}$ = 2.00<br><br><br>$F_{(1,8)}$ = 0.09<br>$F_{(1,8)}$ = 0.62<br>$F_{(1,8)}$ = 8.55	 <br>0.047<br>0.12<br><br><br>0.77<br>0.45<br>0.01

<table>
<tr><td>4K</td><td>Decoding accuracy
vs chance
choice
value
context</td><td>9 sessions from 3 mice</td><td>

Paired t-test</td><td>

$t_{(8)} = 5.33$
$t_{(8)} = 6.65$
$t_{(8)} = 6.13$</td><td>

0.0007
0.0002
0.0003</td></tr>
<tr><td>4M</td><td>Decoding accuracy
vs chance
shuffle
test</td><td>

4 sessions from 2 mice
(ses. with > 100 neurons)</td><td>

Paired t-test</td><td>

$t_{(3)} = 1.06$
$t_{(3)} = 3.49$</td><td>

0.37
0.03</td></tr>
<tr><td>S1D</td><td>p incongruent trials
x switch</td><td>10 mice</td><td>Repeated-measures ANOVA</td><td>$F_{(14,126)} = 7.98$</td><td>6.1×10^{-12}</td></tr>
<tr><td>S1E</td><td>p incongruent choice
consistent with Q vs SI</td><td>10 mice</td><td>Paired t-test</td><td>$t_{(9)} = 5.83$</td><td>0.0003</td></tr>
<tr><td>S6B</td><td>Difference between mouse
and prediction from Q or SI
Sham vs lesion
(log transformed)</td><td>SI

Q</td><td>Paired t-test

Paired t-test</td><td>$t_{(7.7)} = 4.41$

$t_{(42)} = 2.67$</td><td>0.003

0.011</td></tr>
<tr><td>S6D</td><td>p SI consistent after switch:
sham vs lesion</td><td>Sham: 25 sessions from
3 mice
Lesion: 35 sessions from
5 mice</td><td>t-test</td><td>$t_{(42)} = 2.44$</td><td>0.019</td></tr>
<tr><td>S6D</td><td>p Q consistent after switch:
sham vs lesion</td><td>Sham: 25 sessions from
3 mice
Lesion: 35 sessions from
5 mice</td><td>t-test</td><td>$t_{(42)} = 1.55$</td><td>0.13</td></tr>
<tr><td>S6E</td><td>Parameters of model fits
sham vs lesion:
γ
c
bias</td><td>Sham: 25 sessions from
3 mice
Lesion: 35 sessions from
5 mice</td><td>

t-test</td><td>

$t_{(4)} = 5.47$
$t_{(4)} = 0.40$
$t_{(4)} = 0.51$</td><td>

0.005
0.71
0.64</td></tr>
<tr><td>S7A</td><td>p correct sham vs lesion</td><td>Sham: 41 sessions
from 5 mice
Lesion: 43 sessions
from 5 mice</td><td>t-test</td><td>$t_{(8)} = 0.36$</td><td>0.72</td></tr>
<tr><td>S7B</td><td>Difference between mouse
and prediction from Q vs SI
Sham vs lesion</td><td>Sham: 41 sessions
from 5 mice
Lesion: 43 sessions
from 5 mice</td><td>t-test</td><td>$t_{(4)} = 26.32$

$t_{(4)} = 31.93$</td><td>0.00001

0.00001</td></tr>
</table>

S7C	p SI consistent:<br>sham vs lesion	Sham: 41 sessions<br>from 5 mice<br>Caspase: 43 sessions<br>from 5 mice	t-test	$t_{(8)} = 0.20$	0.85

Table S2. Summary of all model augmentations.

Summary of augmentations for each model. Rows of exemplar models of each class, used for further comparisons throughout the manuscript are highlighted. Corresponds to chart in fig. S3B.

Model	bias	stickiness	RP	different α	same α	forget gradual	forget reset	fixed d
random	X							
choice kernel		X						
WSLS								
Q								
Q			X					
Q	X		X					
Q		X	X					
Q	X	X	X					
Q	X							
Q		X						
Dynamic Q								
Dynamic Q			X					
Dynamic Q	X		X					
Dynamic Q		X	X					
Dynamic Q	X							
Dynamic Q		X						
Forgetting Q			X			X		
Forgetting Q	X		X			X		
Forgetting Q			X				X	
Forgetting Q	X		X				X	
Forgetting Q						X		
Forgetting Q	X					X		
Forgetting Q							X	
Forgetting Q	X						X	
Counterfactual Q				X				
Counterfactual Q			X	X				
Counterfactual Q	X		X	X				

Model	bias	stickiness	RP	different α	same α	forget to mean	forget reset	fixed d
Counterfactual Q			X		X			
Counterfactual Q	X		X		X			
Counterfactual Q		X	X	X				
Counterfactual Q		X	X		X			
Counterfactual Q	X			X				
Counterfactual Q					X			
Counterfactual Q	X				X			
Counterfactual Q		X		X				
Counterfactual Q		X			X			
SI	X		X					
SI	X		X					X
SI			X					X
SI			X					
SI	X	X	X					
SI	X	X	X					X
SI		X	X					X
SI		X	X					
SI	X							
SI								
SI		X						

Table S3. Summary of exemplar models.

Model	**Parameters** (mean ± SEM)	**BIC** (mean ± SEM)
Random	$bias$: 0.48 ± 0.01	432.98 ± 9.13
Choice kernel	α: 0.6 ± 0.06<br>β: 4.88 ± 0.04	424.24 ± 8.55
WSLS	ε: 0.73 ± 0.03	411.3 ± 8.25
Q	α_r: 0.7 ± 0.05<br>α_{ur}: 0.53 ± 0.03<br>β: 4.3 ± 0.34<br>$bias$: -0.04 ± 0.04	278.21 ± 12.2
Dynamic Q	α_0: 0.57 ± 0.06<br>κ_r: 0.86 ± 0.03<br>κ_{ur}: 0.74 ± 0.02<br>γ: 0.35 ± 0.05<br>β: 4.46 ± 0.41<br>$bias$: -0.03 ± 0.04	292.1 ± 12.08
Forgetting Q	α_r: 0.7 ± 0.07<br>α_{ur}: 0.67 ± 0.05<br>δ: 0.19 ± 0.05<br>β: 4.5 ± 0.32<br>$bias$: -0.02 ± 0.03	278.19 ± 9.35
Counterfactual Q	α_r: 0.8 ± 0.04<br>α_{ur}: 0.14 ± 0.02<br>β: 2.59 ± 0.17<br>$bias$: -0.02 ± 0.04	267.92 ± 9.15
SI	c: 0.62 ± 0.07<br>d: 0 (fixed)<br>γ: 0.49 ± 0.02<br>$bias$: -0.003 ± 0.008	237.6 ± 10

References and Notes

1. A. M. Wikenheiser, G. Schoenbaum, Over the river, through the woods: Cognitive maps in the hippocampus and orbitofrontal cortex. *Nat. Rev. Neurosci.* **17**, 513–523 (2016). doi:10.1038/nrn.2016.56 Medline

2. S. J. Gershman, N. Uchida, Believing in dopamine. *Nat. Rev. Neurosci.* **20**, 703–714 (2019). doi:10.1038/s41583-019-0220-7 Medline

3. R. C. Wilson, Y. K. Takahashi, G. Schoenbaum, Y. Niv, Orbitofrontal cortex as a cognitive map of task space. *Neuron* **81**, 267–279 (2014). doi:10.1016/j.neuron.2013.11.005 Medline

4. V. D. Costa, V. L. Tran, J. Turchi, B. B. Averbeck, Reversal learning and dopamine: A Bayesian perspective. *J. Neurosci.* **35**, 2407–2416 (2015). doi:10.1523/JNEUROSCI.1989-14.2015 Medline

5. M. K. Eckstein, S. L. Master, R. E. Dahl, L. Wilbrecht, A. G. E. Collins, Reinforcement learning and Bayesian inference provide complementary models for the unique advantage of adolescents in stochastic reversal. *Dev. Cogn. Neurosci.* **55**, 101106 (2022). doi:10.1016/j.dcn.2022.101106 Medline

6. B. M. Babayan, N. Uchida, S. J. Gershman, Belief state representation in the dopamine system. *Nat. Commun.* **9**, 1891 (2018). doi:10.1038/s41467-018-04397-0 Medline

7. C. K. Starkweather, B. M. Babayan, N. Uchida, S. J. Gershman, Dopamine reward prediction errors reflect hidden-state inference across time. *Nat. Neurosci.* **20**, 581–589 (2017). doi:10.1038/nn.4520 Medline

8. P. Vertechi, E. Lottem, D. Sarra, B. Godinho, I. Treves, T. Quendera, M. N. Oude Lohuis, Z. F. Mainen, Inference-based decisions in a hidden state foraging task: Differential contributions of prefrontal cortical areas. *Neuron* **106**, 166–176.e6 (2020). doi:10.1016/j.neuron.2020.01.017 Medline

9. A. J. Qü, L.-H. Tai, C. D. Hall, E. M. Tu, M. K. Eckstein, K. Mishchanchuk, W. C. Lin, J. B. Chase, A. F. MacAskill, A. G. E. Collins, S. J. Gershman, L. Wilbrecht, Nucleus accumbens dopamine release reflects Bayesian inference during instrumental learning. bioRxiv 2023.11.10.566306 [Preprint] (2024); https://doi.org/10.1101/2023.11.10.566306.

10. A. Lak, K. Nomoto, M. Keramati, M. Sakagami, A. Kepecs, Midbrain dopamine neurons signal belief in choice accuracy during a perceptual decision. *Curr. Biol.* **27**, 821–832 (2017). doi:10.1016/j.cub.2017.02.026 Medline

11. R. A. Adams, Q. J. M. Huys, J. P. Roiser, Computational Psychiatry: Towards a mathematically informed understanding of mental illness. *J. Neurol. Neurosurg. Psychiatry* **87**, 53–63 (2016). Medline

12. F. Schlagenhauf, Q. J. M. Huys, L. Deserno, M. A. Rapp, A. Beck, H.-J. Heinze, R. Dolan, A. Heinz, Striatal dysfunction during reversal learning in unmedicated schizophrenia patients. *Neuroimage* **89**, 171–180 (2014). doi:10.1016/j.neuroimage.2013.11.034 Medline

13. Q. J. Huys, D. A. Pizzagalli, R. Bogdan, P. Dayan, Mapping anhedonia onto reinforcement learning: A behavioural meta-analysis. *Biol. Mood Anxiety Disord.* **3**, 12 (2013). doi:10.1186/2045-5380-3-12 Medline

14. A. Mkrtchian, J. Aylward, P. Dayan, J. P. Roiser, O. J. Robinson, Modeling avoidance in mood and anxiety disorders using reinforcement learning. *Biol. Psychiatry* **82**, 532–539 (2017). doi:10.1016/j.biopsych.2017.01.017 Medline

15. A. Radulescu, Y. Niv, State representation in mental illness. *Curr. Opin. Neurobiol.* **55**, 160–166 (2019). doi:10.1016/j.conb.2019.03.011 Medline

16. J. O'Keefe, L. Nadel, *The Hippocampus as a Cognitive Map* (Oxford Univ. Press, 1978).

17. T. Hartley, C. Lever, N. Burgess, J. O'Keefe, Space in the brain: How the hippocampal formation supports spatial cognition. *Philos. Trans. R. Soc. London Ser. B* **369**, 20120510 (2013). doi:10.1098/rstb.2012.0510 Medline

18. P. Andersen, R. Morris, D. Amaral, T. Bliss, J. O'Keefe, Eds., *The Hippocampus Book* (Oxford Univ. Press, 2006).

19. R. W. Komorowski, C. G. Garcia, A. Wilson, S. Hattori, M. W. Howard, H. Eichenbaum, Ventral hippocampal neurons are shaped by experience to represent behaviorally relevant contexts. *J. Neurosci.* **33**, 8079–8087 (2013). doi:10.1523/JNEUROSCI.5458-12.2013 Medline

20. D. M. Bannerman, R. Sprengel, D. J. Sanderson, S. B. McHugh, J. N. P. Rawlins, H. Monyer, P. H. Seeburg, Hippocampal synaptic plasticity, spatial memory and anxiety. *Nat. Rev. Neurosci.* **15**, 181–192 (2014). doi:10.1038/nrn3677 Medline

21. S. Ciocchi, J. Passecker, H. Malagon-Vina, N. Mikus, T. Klausberger, Selective information routing by ventral hippocampal CA1 projection neurons. *Science* **348**, 560–563 (2015). doi:10.1126/science.aaa3245 Medline

22. J. C. Jimenez, K. Su, A. R. Goldberg, V. M. Luna, J. S. Biane, G. Ordek, P. Zhou, S. K. Ong, M. A. Wright, L. Zweifel, L. Paninski, R. Hen, M. A. Kheirbek, Anxiety cells in a hippocampal-hypothalamic circuit. *Neuron* **97**, 670–683.e6 (2018). doi:10.1016/j.neuron.2018.01.016 Medline

23. M. S. Fanselow, H.-W. Dong, Are the dorsal and ventral hippocampus functionally distinct structures? *Neuron* **65**, 7–19 (2010). doi:10.1016/j.neuron.2009.11.031 Medline

24. J. W. Rudy, P. Matus-Amat, The ventral hippocampus supports a memory representation of context and contextual fear conditioning: Implications for a unitary function of the hippocampus. *Behav. Neurosci.* **119**, 154–163 (2005). doi:10.1037/0735-7044.119.1.154 Medline

25. H. Sanders, M. A. Wilson, S. J. Gershman, Hippocampal remapping as hidden state inference. *eLife* **9**, e51140 (2020). doi:10.7554/eLife.51140 Medline

26. H. S. Courellis, J. Minxha, A. R. Cardenas, D. L. Kimmel, C. M. Reed, T. A. Valiante, C. D. Salzman, A. N. Mamelak, S. Fusi, U. Rutishauser, Abstract representations emerge in human hippocampal neurons during inference. *Nature* **632**, 841–849 (2024). doi:10.1038/s41586-024-07799-x Medline

27. L.-H. Tai, A. M. Lee, N. Benavidez, A. Bonci, L. Wilbrecht, Transient stimulation of distinct subpopulations of striatal neurons mimics changes in action value. *Nat. Neurosci.* **15**, 1281–1289 (2012). doi:10.1038/nn.3188 Medline

28. N. F. Parker, C. M. Cameron, J. P. Taliaferro, J. Lee, J. Y. Choi, T. J. Davidson, N. D. Daw, I. B. Witten, Reward and choice encoding in terminals of midbrain dopamine neurons depends on striatal target. *Nat. Neurosci.* **19**, 845–854 (2016). doi:10.1038/nn.4287 Medline

29. R. Hattori, B. Danskin, Z. Babic, N. Mlynaryk, T. Komiyama, Area-specificity and plasticity of history-dependent value coding during learning. *Cell* **177**, 1858–1872.e15 (2019). doi:10.1016/j.cell.2019.04.027 Medline

30. C. C. Beron, S. Q. Neufeld, S. W. Linderman, B. L. Sabatini, Mice exhibit stochastic and efficient action switching during probabilistic decision making. *Proc. Natl. Acad. Sci. U.S.A.* **119**, e2113961119 (2022). doi:10.1073/pnas.2113961119 Medline

31. S. M. Groman, C. Keistler, A. J. Keip, E. Hammarlund, R. J. DiLeone, C. Pittenger, D. Lee, J. R. Taylor, Orbitofrontal circuits control multiple reinforcement-learning processes. *Neuron* **103**, 734–746.e3 (2019). doi:10.1016/j.neuron.2019.05.042 Medline

32. A. Lak, M. Okun, M. M. Moss, H. Gurnani, K. Farrell, M. J. Wells, C. B. Reddy, A. Kepecs, K. D. Harris, M. Carandini, Dopaminergic and prefrontal basis of learning from sensory confidence and reward value. *Neuron* **105**, 700–711.e6 (2020). doi:10.1016/j.neuron.2019.11.018 Medline

33. T. Patriarchi, J. R. Cho, K. Merten, M. W. Howe, A. Marley, W.-H. Xiong, R. W. Folk, G. J. Broussard, R. Liang, M. J. Jang, H. Zhong, D. Dombeck, M. von Zastrow, A. Nimmerjahn, V. Gradinaru, J. T. Williams, L. Tian, Ultrafast neuronal imaging of dopamine dynamics with designed genetically encoded sensors. *Science* **360**, eaat4422 (2018). doi:10.1126/science.aat4422 Medline

34. T. E. J. Behrens, T. H. Muller, J. C. R. Whittington, S. Mark, A. B. Baram, K. L. Stachenfeld, Z. Kurth-Nelson, What is a cognitive map? Organizing knowledge for flexible behavior. *Neuron* **100**, 490–509 (2018). doi:10.1016/j.neuron.2018.10.002 Medline

35. É. Duvelle, R. M. Grieves, M. A. A. van der Meer, Temporal context and latent state inference in the hippocampal splitter signal. *eLife* **12**, e82357 (2023). doi:10.7554/eLife.82357 Medline

36. S. J. Gershman, D. M. Blei, Y. Niv, Context, learning, and extinction. *Psychol. Rev.* **117**, 197–209 (2010). doi:10.1037/a0017808 Medline

37. J. L. Kubie, E. R. J. Levy, A. A. Fenton, Is hippocampal remapping the physiological basis for context? *Hippocampus* **30**, 851–864 (2020). doi:10.1002/hipo.23160 Medline

38. M. C. Fuhs, D. S. Touretzky, Context learning in the rodent hippocampus. *Neural Comput.* **19**, 3173–3215 (2007). doi:10.1162/neco.2007.19.12.3173 Medline

39. M. J. Sharpe, A. M. Wikenheiser, Y. Niv, G. Schoenbaum, The state of the orbitofrontal cortex. *Neuron* **88**, 1075–1077 (2015). doi:10.1016/j.neuron.2015.12.004 Medline

40. J. Zhou, C. Jia, M. Montesinos-Cartagena, M. P. H. Gardner, W. Zong, G. Schoenbaum, Evolving schema representations in orbitofrontal ensembles during learning. *Nature* **590**, 606–611 (2021). doi:10.1038/s41586-020-03061-2 Medline

41. B. F. Sadacca, H. M. Wied, N. Lopatina, G. K. Saini, D. Nemirovsky, G. Schoenbaum, Orbitofrontal neurons signal sensory associations underlying model-based inference in a sensory preconditioning task. *eLife* **7**, e30373 (2018). doi:10.7554/eLife.30373 Medline

42. C. K. Starkweather, S. J. Gershman, N. Uchida, The medial prefrontal cortex shapes dopamine reward prediction errors under state uncertainty. *Neuron* **98**, 616–629.e6 (2018). doi:10.1016/j.neuron.2018.03.036 Medline

43. J. Zhou, M. Montesinos-Cartagena, A. M. Wikenheiser, M. P. H. Gardner, Y. Niv, G. Schoenbaum, Complementary task structure representations in hippocampus and orbitofrontal cortex during an odor sequence task. *Curr. Biol.* **29**, 3402–3409.e3 (2019). doi:10.1016/j.cub.2019.08.040 Medline

44. A. M. Wikenheiser, Y. Marrero-Garcia, G. Schoenbaum, Suppression of ventral hippocampal output impairs integrated orbitofrontal encoding of task structure. *Neuron* **95**, 1197–1207.e3 (2017). doi:10.1016/j.neuron.2017.08.003 Medline

45. R. W. S. Wee, A. F. MacAskill, Biased connectivity of brain-wide inputs to ventral subiculum output neurons. *Cell Rep.* **30**, 3644–3654.e6 (2020). doi:10.1016/j.celrep.2020.02.093 Medline

46. C. Sánchez-Bellot, R. AlSubaie, K. Mishchanchuk, R. W. S. Wee, A. F. MacAskill, Two opposing hippocampus to prefrontal cortex pathways for the control of approach and avoidance behaviour. *Nat. Commun.* **13**, 339 (2022). doi:10.1038/s41467-022-27977-7 Medline

47. R. AlSubaie, R. W. S. Wee, A. Ritoux, K. Mishchanchuk, J. Passlack, D. Regester, A. F. MacAskill, Control of parallel hippocampal output pathways by amygdalar long-range inhibition. *eLife* **10**, e74758 (2021). doi:10.7554/eLife.74758 Medline

48. T. A. LeGates, M. D. Kvarta, J. R. Tooley, T. C. Francis, M. K. Lobo, M. C. Creed, S. M. Thompson, Reward behaviour is regulated by the strength of hippocampus-nucleus accumbens synapses. *Nature* **564**, 258–262 (2018). doi:10.1038/s41586-018-0740-8 Medline

49. S. B. Floresco, C. L. Todd, A. A. Grace, Glutamatergic afferents from the hippocampus to the nucleus accumbens regulate activity of ventral tegmental area dopamine neurons. *J. Neurosci.* **21**, 4915–4922 (2001). doi:10.1523/JNEUROSCI.21-13-04915.2001 Medline

50. A. F. MacAskill, K. Mishchanchuk, Hidden state inference requires abstract contextual representations in the ventral hippocampus, version 1, Zenodo (2024); https://doi.org/10.5281/zenodo.13913194.

51. R. W. S. Wee, K. Mishchanchuk, R. AlSubaie, T. W. Church, M. G. Gold, A. F. MacAskill, Internal-state-dependent control of feeding behavior via hippocampal ghrelin signaling. *Neuron* **112**, 288–305.e7 (2024). doi:10.1016/j.neuron.2023.10.016 Medline

52. S. L. Resendez, J. H. Jennings, R. L. Ung, V. M. K. Namboodiri, Z. C. Zhou, J. M. Otis, H. Nomura, J. A. McHenry, O. Kosyk, G. D. Stuber, Visualization of cortical, subcortical

and deep brain neural circuit dynamics during naturalistic mammalian behavior with head-mounted microscopes and chronically implanted lenses. *Nat. Protoc.* **11**, 566–597 (2016). doi:10.1038/nprot.2016.021 Medline

53. B. Lau, P. W. Glimcher, Dynamic response-by-response models of matching behavior in rhesus monkeys. *J. Exp. Anal. Behav.* **84**, 555–579 (2005). doi:10.1901/jeab.2005.110-04 Medline

54. R. C. Wilson, A. G. Collins, Ten simple rules for the computational modeling of behavioral data. *eLife* **8**, e49547 (2019). doi:10.7554/eLife.49547 Medline

55. Y. Jeong, N. Huh, J. Lee, I. Yun, J. W. Lee, I. Lee, M. W. Jung, Role of the hippocampal CA1 region in incremental value learning. *Sci. Rep.* **8**, 9870 (2018). doi:10.1038/s41598-018-28176-5 Medline

56. A. N. Hampton, R. Adolphs, M. J. Tyszka, J. P. O'Doherty, Contributions of the amygdala to reward expectancy and choice signals in human prefrontal cortex. *Neuron* **55**, 545–555 (2007). doi:10.1016/j.neuron.2007.07.022 Medline

57. J. Li, D. Schiller, G. Schoenbaum, E. A. Phelps, N. D. Daw, Differential roles of human striatum and amygdala in associative learning. *Nat. Neurosci.* **14**, 1250–1252 (2011). doi:10.1038/nn.2904 Medline

58. V. D. Costa, O. Dal Monte, D. R. Lucas, E. A. Murray, B. B. Averbeck, Amygdala and ventral striatum make distinct contributions to reinforcement learning. *Neuron* **92**, 505–517 (2016). doi:10.1016/j.neuron.2016.09.025 Medline

59. N. F. Parker, A. Baidya, J. Cox, L. M. Haetzel, A. Zhukovskaya, M. Murugan, B. Engelhard, M. S. Goldman, I. B. Witten, Choice-selective sequences dominate in cortical relative to thalamic inputs to NAc to support reinforcement learning. *Cell Rep.* **39**, 110756 (2022). doi:10.1016/j.celrep.2022.110756 Medline

60. M. Blanco-Pozo, T. Akam, M. E. Walton, Dopamine-independent effect of rewards on choices through hidden-state inference. *Nat. Neurosci.* **27**, 286–297 (2024). doi:10.1038/s41593-023-01542-x Medline

61. Z. Dong, W. Mau, Y. Feng, Z. T. Pennington, L. Chen, Y. Zaki, K. Rajan, T. Shuman, D. Aharoni, D. J. Cai, Minian, an open-source miniscope analysis pipeline. *eLife* **11**, e70661 (2022). doi:10.7554/eLife.70661 Medline

62. P. Zhou, S. L. Resendez, J. Rodriguez-Romaguera, J. C. Jimenez, S. Q. Neufeld, A. Giovannucci, J. Friedrich, E. A. Pnevmatikakis, G. D. Stuber, R. Hen, M. A. Kheirbek, B. L. Sabatini, R. E. Kass, L. Paninski, Efficient and accurate extraction of in vivo calcium signals from microendoscopic video data. *eLife* **7**, e28728 (2018). doi:10.7554/eLife.28728 Medline

63. M. S. Ahmed, J. B. Priestley, A. Castro, F. Stefanini, A. S. Solis Canales, E. M. Balough, E. Lavoie, L. Mazzucato, S. Fusi, A. Losonczy, Hippocampal network reorganization underlies the formation of a temporal association memory. *Neuron* **107**, 283–291.e6 (2020). doi:10.1016/j.neuron.2020.04.013 Medline

64. T. Akam, I. Rodrigues-Vaz, I. Marcelo, X. Zhang, M. Pereira, R. F. Oliveira, P. Dayan, R. M. Costa, The anterior cingulate cortex predicts future states to mediate model-based

action selection. *Neuron* **109**, 149–163.e7 (2021). doi:10.1016/j.neuron.2020.10.013 Medline

65. S. Bernardi, M. K. Benna, M. Rigotti, J. Munuera, S. Fusi, C. D. Salzman, The geometry of abstraction in the hippocampus and prefrontal cortex. *Cell* **183**, 954–967.e21 (2020). doi:10.1016/j.cell.2020.09.031 Medline
